# Sepsis promotes splenic production of a protective platelet pool with high CD40 ligand expression

**DOI:** 10.1172/JCI153920

**Published:** 2022-04-01

**Authors:** Colin Valet, Mélia Magnen, Longhui Qiu, Simon J. Cleary, Kristin M. Wang, Serena Ranucci, Elodie Grockowiak, Rafik Boudra, Catharina Conrad, Yurim Seo, Daniel R. Calabrese, John R. Greenland, Andrew D. Leavitt, Emmanuelle Passegué, Simón Méndez-Ferrer, Filip K. Swirski, Mark R. Looney

**Affiliations:** 1 Department of Medicine, UCSF, San Francisco, California, USA.; 2 Wellcome-MRC Cambridge Stem Cell Institute, Cambridge, United Kingdom.; 3 Department of Dermatology, Brigham and Women’s Hospital and Harvard Medical School, Boston, Massachusetts, USA.; 4 Columbia Stem Cell Initiative, Department of Genetics and Development, Columbia University Irving Medical Center, New York, New York, USA.; 5 NHS Blood and Transplant, Cambridge, United Kingdom.; 6 Cardiovascular Research Institute, Icahn School of Medicine at Mount Sinai, New York, New York, USA.; 7 Department of Laboratory Medicine, UCSF, San Francisco, California, USA.

**Keywords:** Hematology, Stem cells, Hematopoietic stem cells, Innate immunity, Platelets

## Abstract

Platelets have a wide range of functions including critical roles in hemostasis, thrombosis, and immunity. We hypothesized that during acute inflammation, such as in life-threatening sepsis, there are fundamental changes in the sites of platelet production and phenotypes of resultant platelets. Here, we showed during sepsis that the spleen was a major site of megakaryopoiesis and platelet production. Sepsis provoked an adrenergic-dependent mobilization of megakaryocyte-erythrocyte progenitors (MEPs) from the bone marrow to the spleen, where IL-3 induced their differentiation into megakaryocytes (MKs). In the spleen, immune-skewed MKs produced a CD40 ligand^hi^ platelet population with potent immunomodulatory functions. Transfusions of post-sepsis platelets enriched from splenic production enhanced immune responses and reduced overall mortality in sepsis-challenged animals. These findings identify a spleen-derived protective platelet population that may be broadly immunomodulatory in acute inflammatory states such as sepsis.

## Graphical abstract

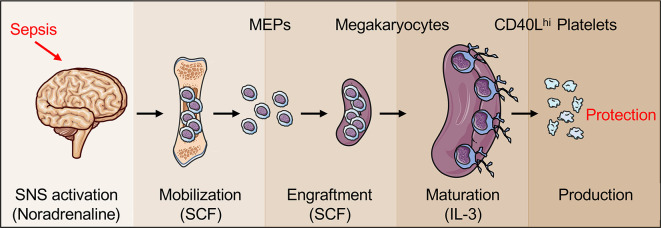

## Introduction

Megakaryocytes (MKs) are the largest hematopoietic cell in the bone marrow (BM) and the progenitors of platelets, which are released locally from the BM, or from intravascular MKs in the lungs for extramedullary platelet biogenesis (1, 2). Platelets are anucleate blood cells that have central roles in hemostasis and arterial thrombosis, the latter representing one of the leading causes of death worldwide (3, 4). The primary physiological role of platelets is to sense vascular injury and adhere and aggregate at vascular lesions to prevent blood loss (3). In addition to the function of platelets in hemostasis, accumulating evidence indicates that platelets play a significant role in the inflammatory response, as they localize at sites of bacterial infection or inflammation (5, 6). Platelets can participate in inflammation via different mechanisms including through interactions with leukocytes and endothelial cells, through release of granule contents and microparticles, and through the synthesis of pro- and antiinflammatory cytokines and surface receptors (5, 7–9).

The BM and extramedullary sites, such as the lung, operate to meet the enormous daily platelet production needs during homeostasis (1, 10). Platelet production and activation are tightly regulated to avoid catastrophic bleeding or arterial occlusion leading to organ failure (10, 11). Megakaryopoiesis is a highly specialized process, whereby hematopoietic BM progenitor cells differentiate into MKs that in turn release platelets into the circulation. Extramedullary hematopoiesis, especially in the spleen, is a common process in a variety of inflammatory conditions (12–15). During acute inflammation, platelet counts may be drastically reduced from a combination of consumption and underproduction (5), while during chronic inflammation, thrombocytosis is common (16). However, during acute inflammation, such as in life-threatening sepsis, it is unknown if there are fundamental changes in the sites of platelet production and resulting platelet phenotypes.

On the basis of our previous work, in which we observed the presence of MK progenitors in the spleen (1), we hypothesized that megakaryopoiesis and platelet biogenesis and function are reshaped during inflammation with a prominent role for splenic production. In this study, we used sepsis as a driver of severe, acute inflammation to study extramedullary megakaryopoiesis through direct imaging of the spleen and lineage-tracing assays enabled by splenic transplantation. We found that sepsis triggered adrenergic-dependent mobilization of hematopoietic precursors from the BM to the spleen, where IL-3 rather than thrombopoietin drove maturation of these cells to immune-skewed MKs. These splenic MKs produced a unique CD40L^hi^ platelet population that showed strong immunomodulatory effects in vitro and in vivo.

## Results

### Sepsis increases splenic megakaryopoiesis and platelet biogenesis.

**Figure 1:**
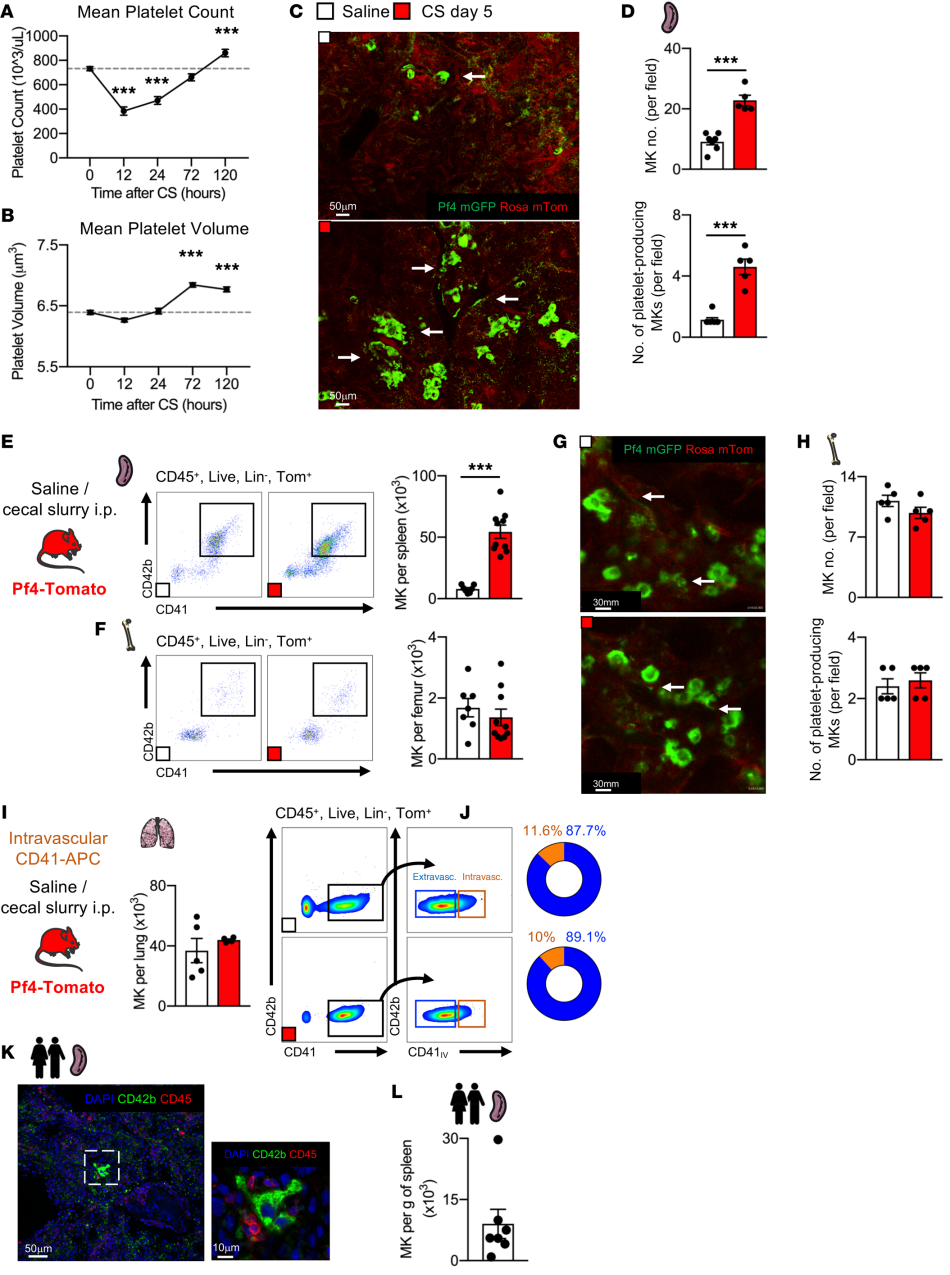
Sepsis induces splenic megakaryopoiesis and platelet production. (**A**) Mean platelet count and (**B**) mean platelet volume after CS injection. *n =* 12 and *n =* 24 mice, respectively. 2PIVM images (**C**) and analysis (**D**) of MK and platelet production (arrows) in spleens from Pf4-mTmG mice 5 days after saline or CS injection. *n =* 7 and *n =* 5 mice, respectively. Scale bars: 50 μm. Enumeration and analysis of MKs in the spleen (**E**) and the BM (**F**) 5 days after saline or CS injection. *n =* 7 and *n =* 10 mice, respectively. 2PIVM images (**G**) and analysis (**H**) of MKs and platelet production (arrows) in BM from Pf4-mTmG mice, 5 days after saline or CS injection. Scale bars: 30 μm. *n =* 5 mice per group. (**I**) Enumeration and analysis of MKs in the lungs 5 days after saline or CS injection. *n =* 5 and *n =* 4 mice, respectively. (**J**) Analysis and frequency of intravascular (intravasc.) and extravascular (extravasc.) MKs in the lungs 5 days after saline or CS injection. *n =* 5 and *n =* 4 mice, respectively. (**K**) Representative images of a MK in a human spleen. Scale bars: 50 μm and 10 μm. (**L**) Enumeration of MKs per gram of human spleen (*n =* 7). Data indicate the mean ± SEM. ***P < 0.0001, by 2-tailed, unpaired Student’s *t* test and 2-way ANOVA.

Sepsis is a life-threatening condition caused by an extreme host response to microbial infection. To investigate the influence of acute inflammation on megakaryopoiesis, we used cecal slurry (CS) injections (17) to model sepsis (Supplemental Figure 1, A–E; supplemental material available online with this article; https://doi.org/10.1172/JCI153920DS1). Thrombocytopenia is a common clinical feature in sepsis (18). Using a nonlethal dose of CS, we observed a sharp decrease in platelet counts followed by a return to normal counts after 5 days (Figure 1A). The platelet rebound was associated with an increased mean platelet volume and an expanded thiazol orange^+^ (RNA-rich, recently produced) platelet population, indicating increased platelet production (Figure 1B and Supplemental Figure 1F). Five days after sepsis induction, when splenomegaly was present (Supplemental Figure 2A), we directly imaged the spleen using 2-photon intravital microscopy (2PIVM) (Supplemental Figure 2B) and fluorescent reporter mice (Pf4-Cre x mTmG, hereafter called Pf4-mTmG) to visualize MKs and platelets (1). We observed a significant increase in MK content and platelet-producing MKs in the spleen (Figure 1, C–E, Supplemental Figure 2, C–E, and Supplemental Video 1), but not in the BM (Figure 1, F–H, Supplemental Figure 2, F and G, and Supplemental Video 2) or the lungs (Figure 1, I and J). Splenic MKs have higher ploidy compared with BM MKs (Supplemental Figure 2, H and I), and sepsis induced a shift toward MKs with higher ploidy in both the BM and spleen (Supplemental Figure 2, H and I). To extend our findings of increased splenic megakaryopoiesis, we used a sterile model of sepsis (i.p. LPS) that resulted in thrombocytopenia, increased platelet production, unchanged BM MK numbers, and increased splenic MKs (Supplemental Figure 2, J–L).

The presence of MKs in the human spleen has not previously been investigated outside the context of extreme extramedullary hematopoiesis (19, 20). We therefore studied human spleen samples collected from organ donors (Table 1). Using immunofluorescence, we observed large, CD42b^+^ cells with polyploid nuclei and proplatelet cytoplasmic extensions in human spleens (Figure 1K and Supplemental Video 3). We confirmed these observations by flow cytometric studies of the human spleens, in which a lineage^–^ (Lin^–^), CD45^+^, CD41a^+^, CD42b^+^ MK population was consistently found (Figure 1L and Supplemental Figure 2M), indicating that the human spleen is a hospitable environment for MK residence.

### Sympathetic nervous system activation regulates MEP mobilization in sepsis.

**Figure 2:**
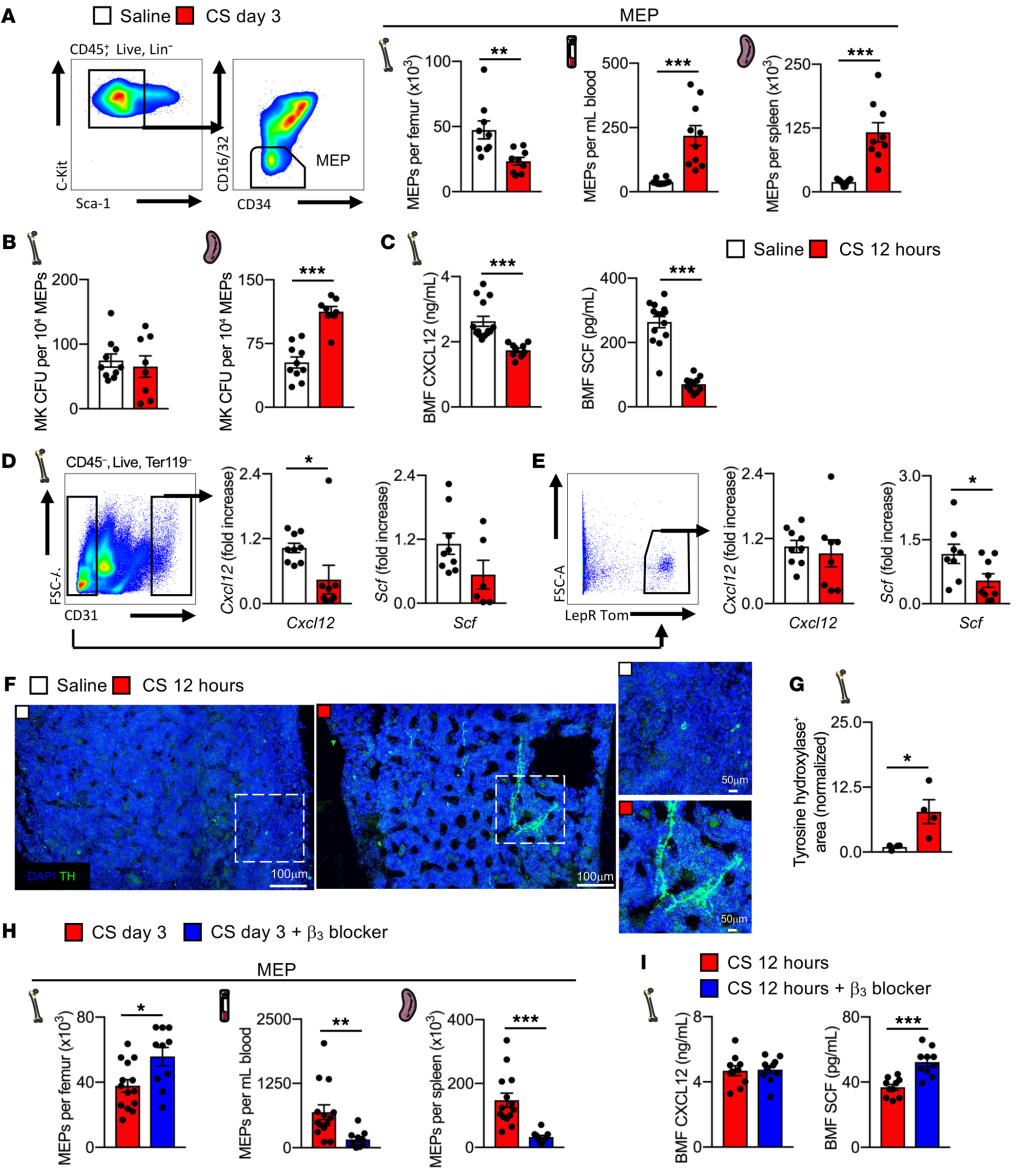
Sepsis induces adrenergic-dependent MEP egress from the BM. (**A**) Flow cytometric analysis of MEPs in the BM, blood, and spleen 3 days after saline or CS injection. *n =* 9 mice per group. (**B**) MegaCult colony-forming assay of BM and spleen MEPs 3 days after saline or CS injection. *n =* 10 and *n =* 8 mice, respectively. (**C**) BM CXCL12 and SCF levels 12 hours after saline or CS injection. *n =* 14 and *n =* 11, respectively. (**D** and **E**) Representative flow sorting of CD31^+^ endothelial and LepR^+^Tom^+^ stromal cells and expression of the retention factors *Cxcl12* and *Scf*, as assessed by quantitative PCR (qPCR) 12 hours after saline or CS injection. (**D**) *n =* 9, *n* = 8 and *n* = 9, *n* = 6 and (**E**) *n* = 9, *n* = 8 and *n* = 8, *n* = 9 mice, respectively. FSC-A, forward scatter area. (**F**) Representative images and (**G**) quantification of tyrosine hydroxylase (TH) staining in the BM 12 hours after saline or CS injection. *n =* 5 and *n =* 4 mice, respectively. Scale bars: 100 μm and 50 μm. (**H**) Flow cytometric analysis of MEPs in the BM, blood, and spleen 3 days after CS injection with or without β3 receptor antagonism. *n =* 14 and *n =* 10 mice, respectively. (**I**) BM CXCL12 and SCF levels 12 hours after CS injection with or without β3 receptor antagonism. *n =* 10 mice per group. Data indicate the mean ± SEM. Significance was assessed using a 2-tailed, unpaired Student’s *t* test. *P < 0.05, **P < 0.005, and ***P < 0.0001. BMF, bone marrow fluid.

To understand the mechanisms responsible for splenic megakaryopoiesis in sepsis, we measured hematopoietic progenitor cells (megakaryocyte-erythrocyte progenitors [MEPs] and their precursors, Lin^–^c-Kit^+^Sca-1^+^ [LSK] cells) in the BM and blood during sepsis (see the gating strategy in Supplemental Figure 3A). MEPs significantly decreased in the BM, even if their proliferation rate was increased (Figure 2A and Supplemental Figure 3B), whereas LSK content increased after sepsis (Supplemental Figure 3D). In contrast, blood and splenic MEPs (Figure 2A and Supplemental Figure 3C) and LSKs (Supplemental Figure 3, E and F) and their proliferation rates increased after sepsis. The capacity of splenic MEPs to differentiate into MK colonies was also increased after sepsis, whereas BM MEP capacities were unaffected (Figure 2B). These results indicate that sepsis triggers an egress of hematopoietic progenitor cells from the BM into the blood with an increase of MEPs in the spleen.

To identify mechanisms regulating sepsis-induced hematopoietic progenitor cell egress, we examined potential roles of retention factors that have been shown to influence the hematopoietic stem cell niche (13, 21, 22). Sepsis decreased the levels of CXCL12 and stem cell factor (SCF) in the BM (Figure 2C), while increasing SCF levels in the blood (Supplemental Figure 3G), which suggests a chemotactic gradient favorable for LSK and MEP egress into the blood. Next, we assessed gene expression of *Scf* and *Cxcl12* in hematopoietic niche stromal cells, endothelial cells, and leptin receptor^+^ (LepR^+^) perivascular cells (13, 21, 22) using LepR-Cre-tdTomato mice. In sepsis, endothelial cells expressed lower *Cxcl12* mRNA (Figure 2D), and LepR^+^ cells expressed lower *Scf* mRNA (Figure 2E). These results are in accordance with a previous study reporting that *Scf* deletion from LepR^+^ cells depleted the BM of MEPs (23, 24).

In homeostasis and in response to stressors, the expression of stem cell retention factors is regulated by the sympathetic nervous system (SNS) through β3-adrenergic receptors, which directly influence the mobilization of hematopoietic progenitors to the blood (14, 25). Indicative of SNS activation, we found increased levels of tyrosine hydroxylase, an enzyme responsible for noradrenaline production in sympathetic fibers, in the BM after sepsis (Figure 2, F and G). Treatment with a β3-adrenergic receptor antagonist increased MEPs and LSKs in the BM, while decreasing their content in the blood and spleen, thus completely reversing these responses during sepsis (Figure 2H and Supplemental Figure 4, A–F). SCF levels in the BM were higher after β3-adrenergic receptor blockade, while CXCL12 levels were unaffected (Figure 2I). These data indicate that sepsis-induced SNS activation decreases the expression of SCF in LepR^+^ stromal cells and induces hematopoietic progenitor cell mobilization from the BM.

### Splenic MEPs originate from the BM.

**Figure 3:**
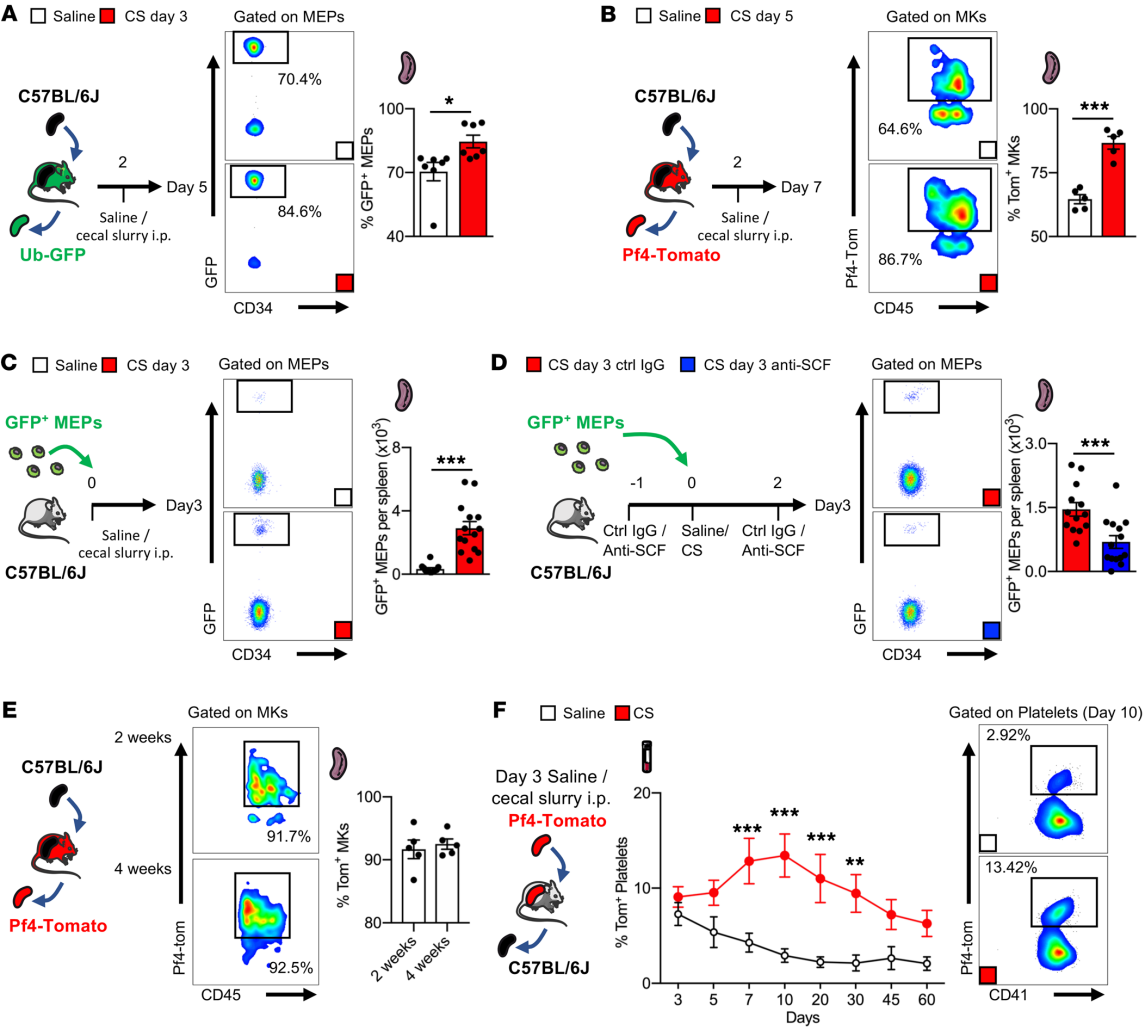
Sepsis promotes BM-derived MK engraftment and platelet production in the spleen. (**A**) Flow cytometric analysis of GFP^+^ MEPs in the spleen 5 days after saline or CS injection. *n =* 7 mice per group. (**B**) Flow cytometric analysis of Tomato^+^ (Tom^+^) MKs in the spleen 5 days after saline or CS injection. *n =* 5 mice per group. (**C**) Flow cytometric analysis of adoptively transferred GFP^+^ MEPs in the spleen 3 days after saline or CS injection. *n =* 12 and *n =* 14 mice, respectively. (**D**) Flow cytometric analysis of adoptively transferred GFP^+^ MEPs in the spleen 3 days after CS injection and treatment with control IgG or SCF-neutralizing antibody. *n =* 13 and *n =* 14 mice, respectively. (**E**) Flow cytometric analysis of Tomato^+^ MKs in the spleen 2 weeks and 4 weeks after transplantation. *n =* 5 mice. (**F**) Percentage of donor-derived CD41^+^, Tomato^+^ platelets. *n =* 7 mice per group. *P < 0.05, **P < 0.005, and ***P < 0.0001, by 2-tailed, unpaired Student’s *t* test (**A**–**D**) or 2-way ANOVA (**F**).

Next, we focused on the mechanisms of MEP enrichment in the spleen during sepsis. Using splenic transplantation to facilitate lineage tracing (Supplemental Figure 5A), we transplanted unlabeled spleens into ubiquitin-GFP mice and analyzed spleen GFP^+^ hematopoietic progenitor engraftment during homeostasis and sepsis. With saline or CS injection 2 days after transplantation and spleen harvesting 5 days after transplantation, 70.4% of splenic MEPs in the control mice were GFP^+^ (i.e., originating from cells outside of the spleen), while 84.6% of splenic MEPs were GFP^+^ following sepsis (Figure 3A). We observed a similar relationship in the GFP^+^ LSK population in the spleen, with 64.2% GFP^+^ LSKs in controls versus 82.1% in septic mice (Supplemental Figure 5B). We used splenic transplantation to also track the origin of splenic MKs. Transplantation of unlabeled spleens into Pf4-tdTomato recipients with or without sepsis revealed that the majority of splenic MKs were derived from cells outside of the spleen and that this increased after sepsis (Figure 3B). Using adoptive transfer of GFP^+^ MEPs into unlabeled mice, we also observed that sepsis increased splenic, but not BM, engraftment of MEPs (Figure 3C and Supplemental Figure 5C).

Depletion of SCF from splenic endothelial or Tcf21^+^ stromal cells can block MEP engraftment in induced extramedullary hematopoiesis (26). We observed that sepsis increased SCF levels in the spleen, suggesting a role for SCF in MEP engraftment (Supplemental Figure 5D). Antibody neutralization of SCF decreased the retention of adoptively transferred GFP^+^ MEPs in the spleen (Figure 3D) but not the BM (Supplemental Figure 5E). By 2 weeks after transplantation of unlabeled spleens into Pf4-tdTomato^+^ recipients, more than 90% of the splenic MKs were tdTomato^+^ (Figure 3E), indicating that the majority of splenic MKs were produced from BM progenitors. Overall, we conclude that in response to sepsis, MEPs exited the BM to engraft in the spleen in an SCF-dependent process.

Next, we used transplantation of Pf4-tdTomato spleens into unlabeled recipient mice to track splenic platelet production. We observed that a control spleen produced approximately 3%–4% of the total circulating platelet pool (tdTomato^+^, CD41^+^ events), and transplantation of spleens obtained 3 days after sepsis significantly increased splenic platelet production to more than 13% of total circulating platelets (Figure 3F). The presence of tdTomato^+^ MKs in the spleen and the absence of tdTomato^+^ MKs in the BM after splenic transplantation confirmed that the observed tdTomato^+^ circulating platelets originated from MKs in the transplanted spleen (Supplemental Figure 5F). We conclude from these results that the spleen is a site of platelet production and that sepsis significantly increases splenic platelet production capacity.

### IL-3 drives splenic megakaryopoiesis.

**Figure 4:**
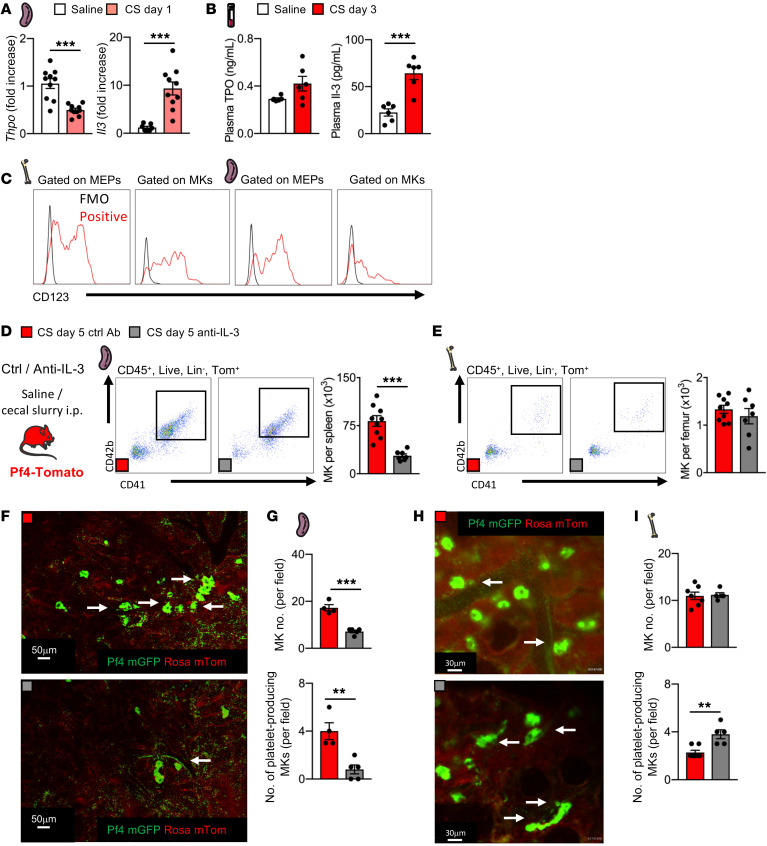
IL-3 drives MK maturation in the spleen during sepsis. (**A**) Expression of *Thpo* and *Il3* in the spleen as assessed by qPCR, 1 day after saline or CS injection. *n =* 10, *n =* 10 and *n* = 7, *n =* 10 mice, respectively. (**B**) Blood levels of TPO and IL-3 after saline injection 3 days after CS injection. *n =* 13, *n =* 9, *n* = 10 and *n* = 10, *n* = 8, and *n* = 9 mice, respectively. (**C**) Surface expression of IL-3Rα (CD123) on MEPs and MKs in the BM (left) and spleen (right). (**D** and **E**) Flow cytometric analysis of MKs in the spleen (**D**) and BM (**E**), 5 days after CS injection and treatment with anti-HRP or anti–IL-3–neutralizing antibody. *n =* 9 and *n =* 7 mice, respectively. Ctrl, control. (**F** and **G**) 2PIVM images (**F**) and analysis (**G**) of MKs and platelet production (arrows) in the spleen in Pf4-mTmG mice 5 days after CS injection and treatment with anti-HRP or anti–IL-3–neutralizing antibody. *n =* 4 and *n =* 5 mice, respectively. Scale bars: 50 μm. (**H** and **I**) 2PIVM images (**H**) and analysis (**I**) of MKs and platelet production (arrows) in the BM in Pf4-mTmG mice 5 days after CS injection and treatment with daily anti-HRP or anti–IL-3 antibody. Scale bars: 30 μm. *n =* 7 and *n =* 5 mice, respectively. Data indicate the mean ± SEM. **P < 0.005 and ***P < 0.0001, by 2-tailed, unpaired Student’s *t* test.

The maturation of BM MEPs into MKs is predominately driven by thrombopoietin (Tpo) (27), however, in the spleen, injections of recombinant IL-3 into WT mice were found to increase the number of splenic MKs by more than 4-fold (28). IL-3 serum levels also significantly increase during sepsis and drive splenic myelopoiesis in atherosclerotic mice (29, 30). Following sepsis, we tested candidate MK growth factors and observed decreased splenic expression of *Tpo*, *Csf2,* and *Ccl5,* while *Il1b* was unchanged and *Il3* and *Il6* were significantly increased (Figure 4A and Supplemental Figure 6A). At the protein level, plasma Tpo concentrations remained unchanged on post-sepsis day 3, while IL-3 was significantly increased (Figure 4B). IL-3 signals through a heterodimer composed of CD123, the IL-3 receptor α chain, and CD131, the IL-3 receptor common β chain. Both MEPs and MKs expressed CD123 in the BM and spleen (Figure 4C). To determine whether IL-3 regulates MK maturation in the spleen during sepsis, we treated septic mice with an IL-3–neutralizing antibody or an isotype control antibody. IL-3 blockade had no impact on the mean platelet count or volume kinetics following sepsis (Supplemental Figure 6, B and C) but reduced the splenic MK content and proplatelet production (Figure 4, D, F, and G, and Supplemental Video 4). Total BM MKs were not affected (Figure 4E), but intravital imaging revealed that IL-3 blockade increased the number of MKs producing platelets in the BM (Figure 4, H and I, and Supplemental Video 5), which could be a compensatory response for the decreased splenic production of platelets. Anti–IL-3 treatment did not affect LSK or MEP numbers, nor did it affect proliferation rates in the BM, blood, or spleen (Supplemental Figure 6, D–H), which suggests that IL-3 blockade specifically prevented splenic MEP differentiation into MKs.

To confirm that IL-3, rather than Tpo, drives splenic megakaryopoiesis, we studied MK and platelet responses to sepsis in mice lacking the Tpo receptor (*c-mpl^–/–^*). As in WT mice, in *c-mpl^–/–^* mice, we observed increased MK numbers in the spleen after sepsis, with no effect of the gene knockout on platelet counts or volume or BM MK content (Supplemental Figure 7, A–C), confirming that Tpo was not driving megakaryopoiesis in the spleen. When IL-3 was neutralized in *c-mpl^–/–^* mice during sepsis, the MK numbers in the spleen returned to the nonseptic baseline, whereas the BM MK content remained unaffected (Supplemental Figure 7, B and C). LSK and MEP populations and proliferation rates in the BM, blood, and spleen of *c-mpl^–/–^* mice, with or without IL-3 blockade, had kinetics similar to that of WT mice during sepsis (Supplemental Figure 7, D–H). We conclude from these results that IL-3 regulates megakaryopoiesis in the spleen during sepsis.

### Splenic production of a CD40L^hi^ platelet population following sepsis.

**Figure 5:**
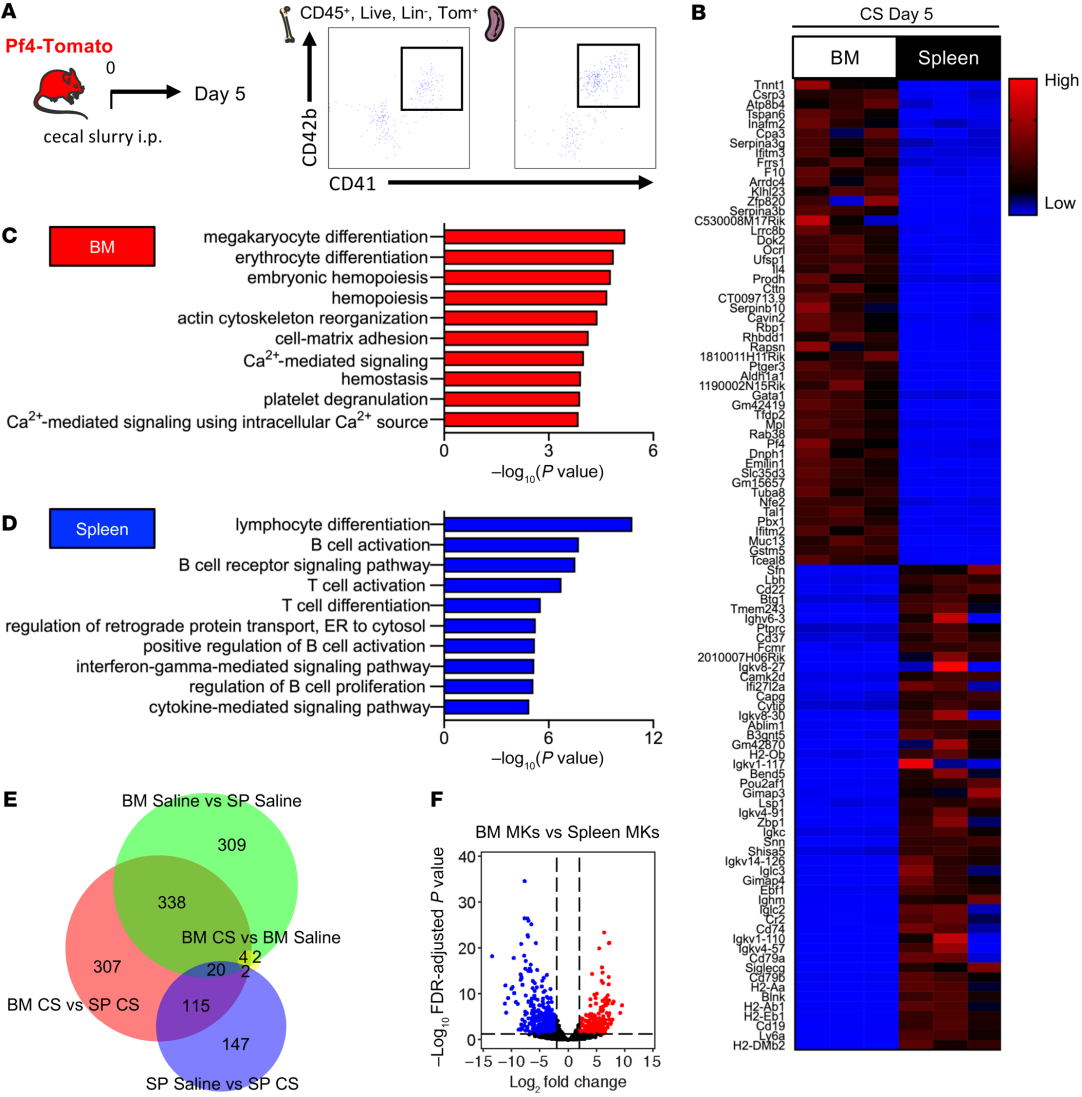
The spleen produces immune-skewed MKs. (**A**) Gating strategy for MKs in the spleen. (**B**) Pf4-Tomato^+^ MKs were sorted from the BM and spleen 5 days after sepsis, followed by mRNA isolation and sequencing. Relative mRNA expression from low (blue) to high (red) of the top 50 genes differentially increased in the BM versus spleen (upper part) and the top 50 genes differentially increased in the spleen versus BM (lower part). (FDR <0.05). (**C** and **D**) GO-BP analysis of genes upregulated in BM MKs (**C**) or upregulated in splenic MKs (**D**) 5 days after CS injection. The top 10 biological processes are shown. (**E**) BioVenn diagram of differentially expressed genes between BM and splenic (SP) MKs 5 days after saline or CS injection. (**F**) Volcano plots of DEG fold change between BM and splenic MKs 5 days after sepsis. *n =* 3 mice.

Previous studies have identified that the tissue of origin significantly influences the transcriptional profile of MKs (1, 2). We used RNA-Seq to characterize and compare BM and splenic MKs with or without sepsis, while carefully excluding contamination from other immune cell populations (CD45^+^, Lin^–^, CD138^–^, CD19^–^, Tom^+^, CD41^+^, CD42b^+^; Figure 5A and Supplemental Figure 8A). While BM MKs expressed classical platelet transcriptional signatures, splenic MKs displayed an immune-like transcriptional signature during homeostasis and in sepsis, indicating that the tissue of residence was the primary driver of changes in MK phenotype (Figure 5, B–D, and Supplemental Figure 8, B–D). We identified low numbers of differentially expressed genes when comparing BM or splenic MKs in saline versus sepsis conditions (Figure 5E). Conversely, we detected a high number of differentially expressed transcripts between BM and splenic MKs during homeostasis (Figure 5E, Supplemental Figure 8E, and Supplemental Table 1) and after sepsis (Figure 5, E and F, and Supplemental Table 2). These results indicate that tissue imprinting, rather than sepsis itself, is the major driver of changes in the MK transcriptome and suggest that the spleen could be capable of producing a functionally distinct population of platelets.

**Figure 6:**
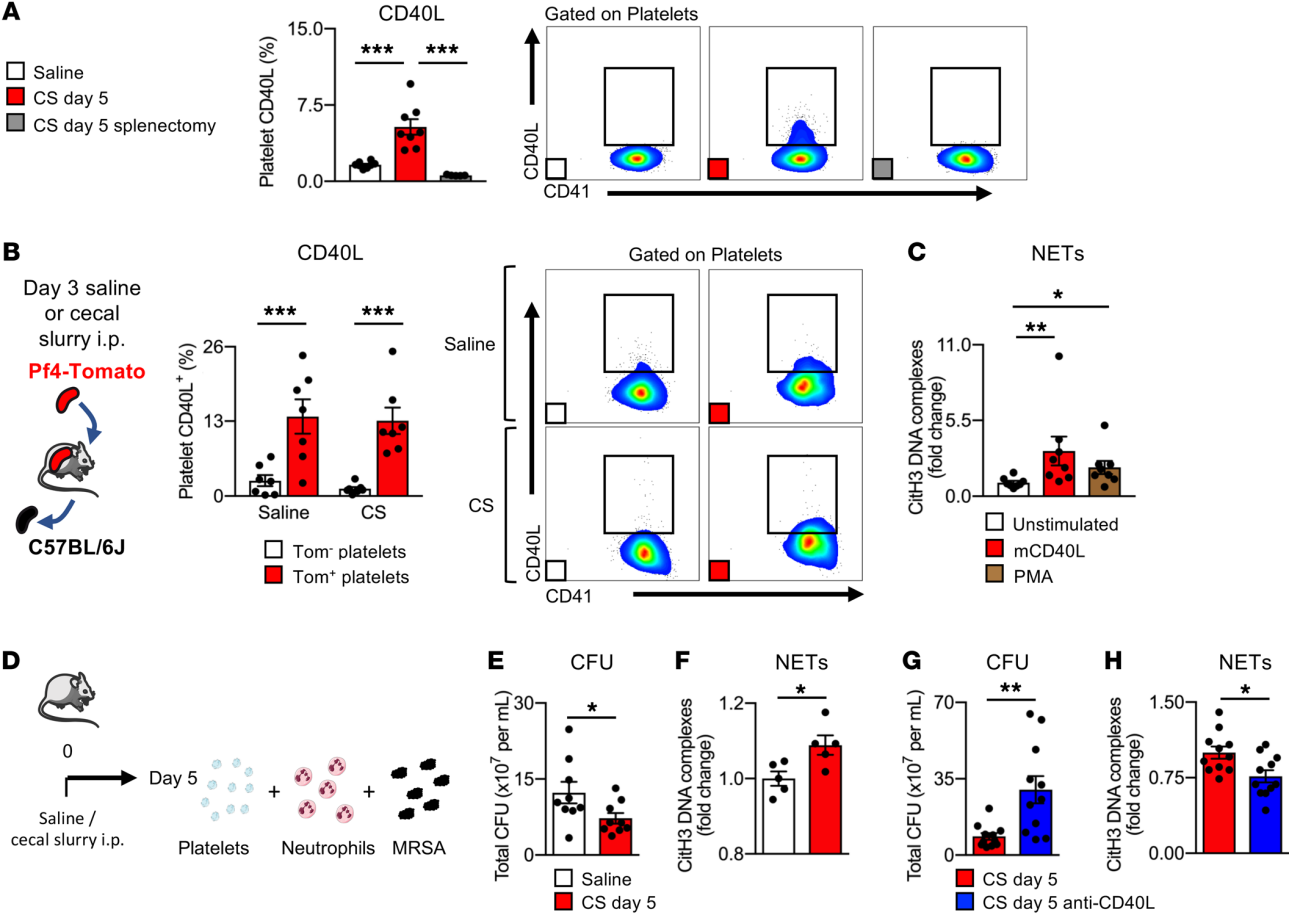
The spleen produces CD40L^hi^ platelets. (**A**) Percentage and analysis of CD40L^+^ platelets 5 days after saline injection, CS injection, or CS injection into splenectomized mice. *n =* 8, *n* = 8, and *n* = 5 mice, respectively. (**B**) Percentage of CD40L^+^ donor-derived Tomato^+^ platelets 10 days after spleen transplantation. *n =* 7 mice per group. (**C**) CitH3-DNA complexes (NETs) after incubation of BM neutrophils with recombinant mouse CD40L or PMA. *n =* 8 mice per group. (**D**) Schematic of platelet, neutrophil, and MRSA coincubation experiments. (**E** and **F**) Bacterial CFU (**E**) and CitH3-DNA complexes (NETs) (**F**) after neutrophil, MRSA, and platelet (collected from saline-injected mice or 5 days after CS injection into mice) coincubation. (**E**) *n =* 10 mice per group and (**F**) *n =* 5 mice per group. (**G** and **H**) Bacterial CFU (**G**) and CitH3-DNA complexes (**H**) after neutrophil, MRSA, and platelet (collected from mice 5 days after CS injection) coincubation with or without treatment with an anti-CD40L–neutralizing antibody. *n =* 11 mice per group. Data indicate the mean ± SEM. *P < 0.05, **P < 0.005, and ***P < 0.0001, by 1-ANOVA (**A** and **C**), 2-way ANOVA (**B**), and 2-tailed, unpaired Student’s *t* test (**E**–**H**).

P-selectin (CD62P) and CD40 ligand (CD40L) are proteins stored in platelet granules, released during activation, and are 2 major pathways used by platelets to interact with leukocytes. On day 5 after sepsis, when platelet counts were back to normal, we observed that, while platelet CD62P surface expression was similar to that in baseline controls, CD40L surface expression was significantly increased (Figure 6A and Supplemental Figure 9A). Splenectomy resulted in the disappearance of this CD40L^hi^ platelet population following sepsis (Figure 6A). To confirm that this CD40L^hi^ platelet population was produced in the spleen, we transplanted Pf4-tdTomato spleens into unlabeled recipient mice. We observed that platelets, originating from the donor spleen sourced from either control mice or from mice 3 days after sepsis, expressed high levels of CD40L compared with platelets originating from the BM (Figure 6B). To rule out platelet activation as the source for CD40L, we tested for surface expression of CD62P but did not find increased expression (Supplemental Figure 9B). These results show that the spleen produced a CD40L^hi^ platelet population and that the overall production of these spleen-derived platelets was increased in sepsis.

CD40L is capable of influencing a variety of immune responses (31), including an association with the release of bactericidal neutrophil extracellular traps (NETs) (32). Using an in vitro NETs assay, we found that recombinant mouse CD40L induced NETs to the same extent as the potent NET inducer PMA (ref. 33 and Figure 6C). We and others have previously found that platelets are potent inducers of NETs (8, 34). Here, when platelets obtained in the resolution phase of sepsis, enriched in CD40L^hi^ platelets, were incubated with neutrophils and live bacteria (methicillin-resistant *Staphylococcus aureus* [MRSA]) (Figure 6D), we observed increased NETs and enhanced bactericidal activity, as indicated by reduced bacterial CFU compared with cocultures of neutrophils and MRSA with control platelets (Figure 6, E and F). This effect was lost when using post-sepsis platelets from splenectomized mice (Supplemental Figure 9, C and D) or with the addition of a neutralizing antibody against CD40L during coincubation (Figure 6, G and H). Taken together, these results indicate that the CD40L^hi^ platelet population produced in increased amounts by the spleen during sepsis has immunomodulatory capabilities to enhance microbial clearance.

### Post-sepsis platelet transfusion protects against sepsis.

**Figure 7:**
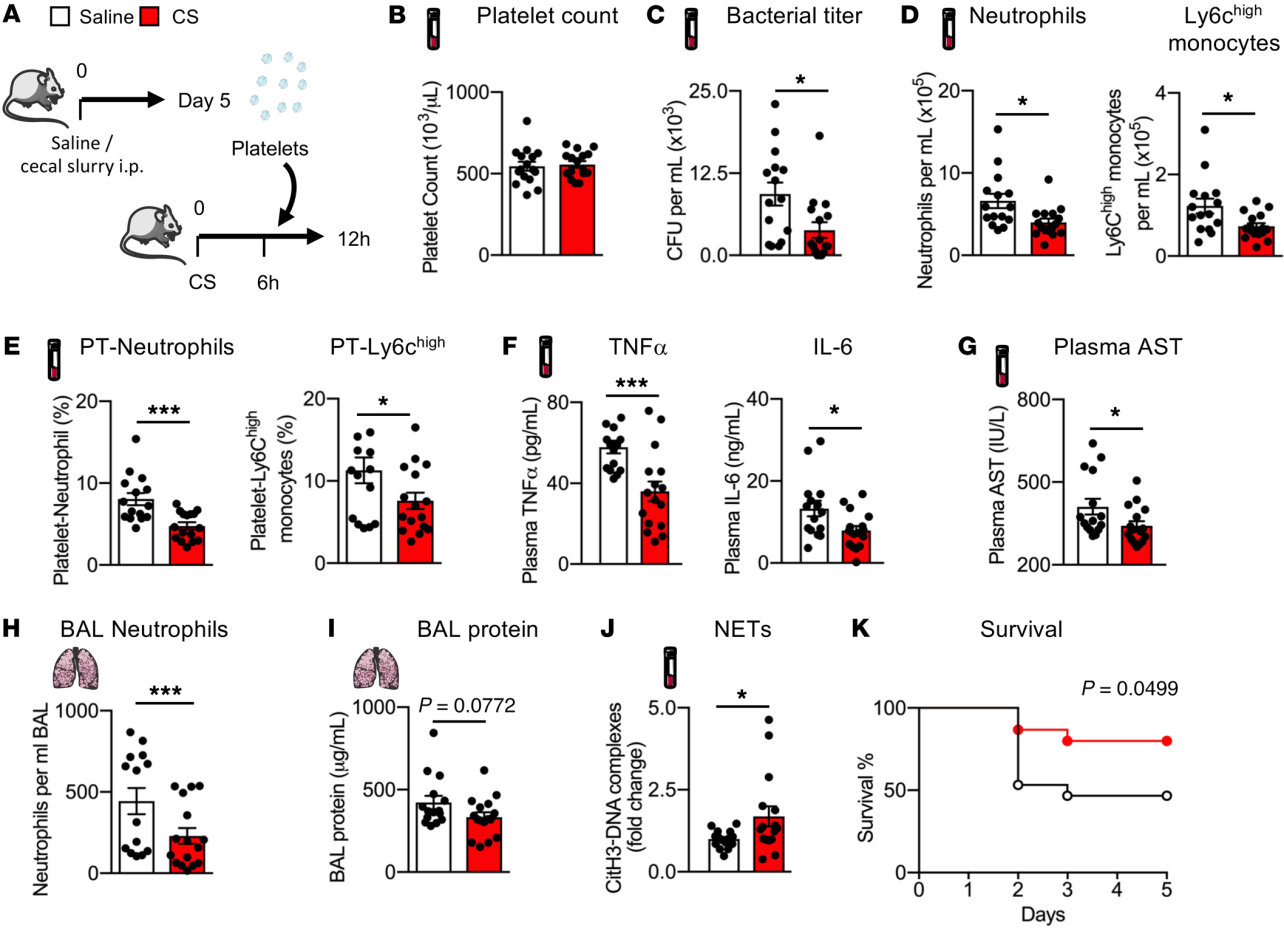
Transfusion of post-sepsis platelets confers protection against sepsis. (**A**) Schematic of septic mice transfused with platelets isolated on day 5 after saline or CS injection experiments. (**B**) Mean platelet counts, (**C**) bacterial CFU in the blood, (**D**) enumeration of neutrophils and Ly6C^hi^ monocytes in the blood, (**E**) enumeration of neutrophil-platelet and Ly6C^hi^ monocyte-platelet aggregates in the blood, (**F**) plasma TNF-α and IL-6 levels, (**G**) plasma AST levels, (**H**) BAL neutrophil count, (**I**) BAL protein levels, and (**J**) CitH3-DNA complexes (NETs) in the blood. *n =* 15–16 mice for all measurements. (**K**) Kaplan-Meier survival curve for mice transfused with 3 × 10^8^ platelets on day 5 after saline or CS injection. *n =* 15 mice per group. Data indicate the mean ± SEM. *P < 0.05 and ***P < 0.0001, by 2-tailed, unpaired Student’s *t* test (**B**–**J**) and Gehan-Breslow-Wilcoxon test (**K**).

There are no precision therapies for sepsis beyond antibiotics and supportive care. A previous study showed that platelet transfusions are potentially protective during septic shock (35). As we found that CD40L^hi^ spleen-derived platelets enhanced bacterial killing, we hypothesized that transfusions enriched with this phenotypically altered platelet pool might be superior to standard platelets as a transfusion therapy for sepsis. To test this hypothesis, we therefore isolated platelets from either control or post-sepsis mice (Supplemental Figure 10A), transfused these washed platelets into WT mice challenged 6 hours previously with CS injections, and then sacrificed the transfused animals 12 hours after CS injection when bacteremia peaks in this model (Figure 7A). Relative to control platelet transfusions, post-sepsis platelet transfusions did not result in altered mean platelet counts or body temperature (Figure 7B and Supplemental Figure 10B), but mice transfused with post-sepsis platelets had a significantly decreased bacterial load in the blood (Figure 7C), while the splenic bacterial load remained unchanged (Supplemental Figure 10C). Treating mice with post-sepsis platelet transfusions also resulted in reductions in several readouts of sepsis-induced inflammation and injury. Transfusions of post-sepsis platelets resulted in reduced blood neutrophil and Ly6C^hi^ monocyte counts (Figure 7D and Supplemental Figure 10D), and a lower proportion of these inflammatory cells aggregated with platelets (Figure 7E and Supplemental Figure 10E). Post-sepsis platelet transfusions also reduced plasma levels of TNF-α and IL-6 (Figure 7F) and the organ injury biomarker aspartate aminotransferase (AST) (Figure 7G). We also detected a reduction in neutrophil migration and a trend toward decreased plasma protein leakage into the lung airspaces (Figure 7, H and I, and Supplemental Figure 10F).

In contrast with other inflammatory mediators and in concordance with our in vitro studies, the levels of plasma NETs were increased following transfusions with post-sepsis platelets (Figure 7J). These results suggest that transfusions enriched in CD40L^hi^ platelets generated after sepsis promote the release of NETs to enable more efficient bacterial containment, which results in reduced inflammation and organ injury. Most important, mice transfused with post-sepsis platelets were protected from death compared with mice transfused with normal platelets (Figure 7K). We conclude from these experiments that transfusions of post-sepsis platelets, enriched in spleen-derived CD40L^hi^ immune-skewed platelets, are strongly immunomodulatory in sepsis.

Since sepsis might significantly modify platelet phenotypes beyond CD40L expression (36), we repeated the platelet transfusion experiments using platelets from *Cd40l^–/–^* mice obtained 5 days after saline or CS injection (Supplemental Figure 11A). We did not observe any differences in blood bacterial load, circulating inflammatory cells, or plasma TNF-α, IL-6, AST, or NET levels (Supplemental Figure 11, B–G). These results suggest that the protective effects observed with post-sepsis platelet transfusions are dependent on the expression of CD40L on platelets.

## Discussion

Platelets are critical immune effectors, and yet we are still learning about the important sites of production and their immune capabilities (5, 9). Sepsis commonly provokes thrombocytopenia, which leads to emergency platelet production to avoid bleeding (37). Although it is known that sepsis induces extramedullary myelopoiesis in the spleen (15, 38), less is known about the dynamics and localization of megakaryopoiesis and platelet production. The presence of MEPs and platelet-producing MKs in the spleen during homeostasis (1) led us to hypothesize that splenic MKs could be essential in emergency platelet production following sepsis-induced thrombocytopenia. Our results indicate that sepsis dramatically increased splenic megakaryopoiesis and platelet production and that splenic MKs and their platelet progeny were unique in both their immune function and their regulation by IL-3.

Until recently, megakaryopoiesis and platelet production were thought to occur solely in the BM, but intravital microscopy studies have revealed platelet-producing MKs in the spleen and lung during homeostasis (1). Our results indicate that sepsis induced an important increase of MKs and their progenitor cells, MEPs, in the spleen, while MEPs in the BM were decreased. In homeostasis or in response to injuries such as myocardial infarction, hematopoietic progenitors are known to exit the BM and relocate to other tissues for extramedullary hematopoiesis (14, 25, 39). This process is in part governed by the SNS, which controls the expression and secretion of retention factors in the BM (21, 22). In line with previous reports (23, 24), we observed that sepsis boosted SNS activity to reduce medullar SCF levels by acting on β3-adrenergic receptors present on the LepR^+^ perivascular cells and, in turn, allowing the release of MEPs into the circulation.

The spleen is a well-characterized extramedullary hematopoietic site (12). In induced extramedullary hematopoiesis assays, splenic endothelial cells and Tcf21-expressing perivascular cells were found to control SCF levels and eventually the splenic engraftment of hematopoietic progenitors (26). Using spleen transplantation and lineage-tracing experiments, we reveal that sepsis enhanced splenic SCF levels and drove BM-released and circulating MEPs to engraft. In the BM, megakaryopoiesis is promoted by thrombopoietin and other cytokines (27). IL-3 is a cytokine known for being greatly increased after sepsis and for enhancing BM myelopoiesis (30). Our results indicate that splenic megakaryopoiesis in response to sepsis was driven by IL-3, not Tpo. Increases in the levels of IL-3, and potentially those of other splenic sources of cytokines, had profound effects on the production of immune-skewed MKs in the spleen. These immune-skewed MKs contributed to 3%–4% of circulating platelets during homeostasis, but with sepsis, the production of this platelet pool rose to greater than 13% of total platelets, with the immune signature of spleen-derived platelets being the expression of surface CD40L. To our knowledge, this is the first description of a circulating platelet subpopulation.

CD40L on platelets participates in immune responses either by direct contact with other cell types or after being shed from the platelet surface (31, 40). We observed that platelets enhanced the release of NETs, which led to more efficient microbial containment, and this effect was dependent on platelet expression of CD40L. Likewise, transfusion of post-sepsis platelets, enriched in splenic production of CD40L, led to improved bacterial clearance (perhaps through increased NETs), reduced organ injury, and improved survival, and this protective effect was lost when platelets were sourced from *Cd40l^–/–^* mice. Taken together, these data strongly implicate the platelet CD40L pathway in protective responses in sepsis, however, other factors might be involved and will be investigated in future studies. Approximately one-third of sepsis survivors are readmitted to the hospital within 90 days (41). The production of an immune-protective platelet pool during sepsis might also represent the host adaptation to fight against post-sepsis syndrome, which is characterized by prolonged immunosuppression and the threat of a secondary infection.

Our findings could inform precision therapy for sepsis through a cellular approach of transfusing platelets that are preprogrammed for immune functions like enhancing microbial clearance directly or through interactions with other immune cells. Because of the increased demand for platelet transfusions and the limitations of blood donation, major progress has been made in the production of platelets in vitro (42). We believe our study represents a first step in the characterization of a unique platelet population with immunoprotective capacities and could add sophistication to these approaches by enabling the production of platelets for specific indications, such as in sepsis.

## Methods

### Animals

Mice were housed and bred under specific pathogen–free conditions at the UCSF Laboratory Animal Research Center. Male and female mice between 8 and 12 weeks of age were used for the experiments. WT C57BL/6J, C57BL/6-Tg(Pf4-icre)Q3Rsko/J, B6.Cg-*Gt(ROSA)26Sor^tm14(CAG-tdTdTomato)Hze^*/J, B6.129(Cg)-*Gt(ROSA)26Sor^tm4(ACTB-tdTdTomato,-EGFP)Luo^*/J, C57BL/6-Tg(UBC-GFP)30Scha/J, B6.129(Cg)-*Lepr^tm2(cre)Rck^*/J and B6.129S2-CD40lg^tm1lmx^/J mice were purchased from The Jackson Laboratory. *c-mpl*^−/−^ mice (on a C57BL/6 background) were obtained from Genentech through a material transfer agreement.

### CS sepsis model

The CS preparation was produced as described previously (17). Briefly, cecal content from C57BL/6J mice was mixed with a solution of PBS and 10% glycerol at a ratio of 1 mL per 100 mg cecal content. The CS was then filtered sequentially through 4 sterile meshes (860, 380, 190, and 74 μm). The CS (200 μL) was administered i.p. for nonlethal sepsis. Mice received daily s.c. injections of 700 μL normal saline solution to alleviate fluid loss during sepsis for 48 hours. The rectal temperature was measured by insertion of a temperature sensor under anesthesia. For survival experiments, the mice were administered 400 μL CS i.p.

### LPS sepsis model

LPS from *Escherichia coli* O111:B4 (10 mg/kg) was administered i.p. to C57BL/6 WT mice to model nonlethal sepsis. Mice received daily s.c. injections of 700 μL normal saline solution for supportive care during sepsis. The mice were euthanized 3 days after LPS.

### In vivo treatments

#### Treatment with β_3_ adrenergic receptor antagonist.

Mice were injected twice daily i.p. with SR59230A, a selective adrenergic β_3_ receptor antagonist obtained from MilliporeSigma, at a dose of 5 mg/kg.

#### Adoptive transfer of GFP^+^ MEPs.

Approximately 50,000 Lin^–^c-Kit^+^Sca-1^–^CD34^–^CD16^–^CD32^–^ MEPs were sorted from UBC-GFP mice and adoptively transferred i.v. into C57BL/6J mice.

#### In vivo Scf neutralization.

C57BL/6J mice were injected i.v. with 300 μg anti-mouse anti-SCF antibody or control goat IgG (R&D Systems) in 300 μL PBS the day before and 2 days after sepsis induction. On the day of sepsis induction, 50,000 sorted GFP^+^ MEPs were adoptively transferred i.v.

#### In vivo IL-3 neutralization.

C57BL/6J, Pf4-Cre-tdTdTomato, or *c-mpl*^−/−^ mice were injected i.p. once daily for 3 or 5 days with either a neutralizing anti–mouse IL-3 antibody (100 mg, clone MP2-8F8, Bio X Cell) or control IgG1 (100 mg, clone HRPN, Bio X Cell).

### Spleen transplantation

The surgical procedure for mouse spleen transplantation was adapted from previously published protocols (43, 44). Briefly, donor mice were anesthetized with an i.p. injection of ketamine (50 mg/kg) and xylazine (10 mg/kg). After ensuring adequate anesthesia with a paw pinch, a midline abdominal incision was made, and the intestines were moved to the right flank of the abdomen to expose the spleen. The spleen was then covered with a piece of sterile gauze soaked with warm saline (37°C) to minimize its damage during surgery. The short gastric vein, superior pancreaticoduodenal vein, and left gastric vessels were ligated with 7-0 silk suture, respectively. The part of the portal vein connecting to the splenic vein was then dissected and mobilized (for the creation of the splenic vein–portal vein patch later). The spleen and the connected pancreatic tissue were flipped to the right flank of the abdomen to expose the splenic artery, the celiac trunk, and the aorta. Heparin (50 units) was then injected into the inferior vena cava (IVC). The abdominal aorta near the celiac trunk was dissected and mobilized (for the creation of the splenic artery–celiac trunk–abdominal aorta patch later). The portal vein distal to the splenic vein and the aorta above the celiac trunk were ligated with a 7-0 suture. The spleen was perfused with 3–4 mL cold heparinized saline (4°C, 100 units/mL) through the abdominal aorta. The splenic artery–celiac trunk–abdominal aorta patch was prepared by transecting the aorta below the celiac trunk. The splenic vein–portal vein patch was prepared by transecting the portal vein proximal to the liver. The spleen graft was then removed and preserved in cold saline (4°C). For the recipient mice, the aorta and IVC were cross-clamped using 2 microvascular clips. The splenic artery–celiac trunk–abdominal aorta patch was anastomosed to the recipient aorta, and the splenic vein–portal vein patch was anastomosed to the recipient IVC using 11-0 sutures. The microvascular clips were removed to perfuse the donor spleen. The native splenic artery and vein were occluded with a 7-0 suture, and the recipient native spleen was removed. The abdomen was closed with a 6-0 suture. Mice received buprenorphine (0.1 mg/kg) every 12 hours for 2 days after the operation. The duration of ischemia of the spleen graft ranged from 30–35 minutes.

### Splenectomy

The procedure for mouse splenectomy was as follows. The mouse was placed on the surgical platform with its left side facing up after anesthesia via ketamine (50 mg/kg) and xylazine (10 mg/kg). The fur on the left side was shaved, and the skin was wiped with an antiseptic povidone-iodine pad and a 70% ethanol pad. A 2 cm horizontal skin incision midway between the last rib and the hip joint was made with surgical scissors. After loosening the connective tissue, a 1 cm incision was made in the peritoneal wall. The spleen was then gently pulled out with forceps. The splenic artery and vein were ligated with a 7-0 silk suture, and the spleen was removed. The peritoneal wall was closed with 1 absorbable 5-0 silk suture, and the skin was closed with two 5-0 silk sutures. Mice received buprenorphine (0.1 mg/kg) every 12 hours for 2 days after the operation. The mice were injected with saline or CS seven days after the splenectomy.

### Cell collection

BM cells were collected by flushing bones with PBS before passing through a 26 gauge needle to create a single-cell suspension, and RBCs were lysed with RBC lysis buffer (BioLegend). Spleens were crushed onto either a 40 μm or 100 μm filter, followed by RBC lysis. Lungs were placed in 2 mL PBS with 5 μg/mL DNaseI (Roche) and 0.5 mg/mL Liberase (Roche), minced with scissors in 15 mL tubes, and digested for 30 minutes at 37°C before filtration through a 100 μm cell strainer and RBC lysis. Peripheral blood was collected by retro-orbital bleeding or vena cava puncture before RBC lysis. For BM stromal cell isolation, BM was digested in digestion mix (1 mg/mL collagenase IV, MilliporeSigma, C5138), 2 mg/mL dispase (Gibco, Life Technologies, Thermo Fisher Scientific, 17105-041), and 5 μg/mL DNase I (Thermo Fisher Scientific, 90083) in HBSS buffer (Gibco, Life Technologies, Thermo Fisher Scientific, 14025-092), 3 times for 15 minutes at 37°C to isolate LepR^+^ and endothelial cells.

### Flow cytometry

Single-cell suspensions were stained in FACS buffer (0.5% BSA and 2 mM EDTA in PBS). The following anti-mouse antibodies were used for flow cytometry: anti-CD41 (BioLegend, clone MWReg30, lot B203702); CD42b (Emfret, clone Xia.G5, lot 0401-D); anti-CD45 (BioLegend, clone 30-F11, lot B294297); anti-CD3 (BioLegend, clone 17A2, lot B274725); anti-NK1.1 (BioLegend, clone PK136, lot B241353); anti-CD11b (BioLegend, clone M1/70, B279987); anti-F4/80 (BioLegend, clone BM8, lot B280040); anti-B220 (BioLegend, clone RA3-6B2, lot B280298); anti-CD31 (BD Biosciences, clone 390, lot 9140841); anti-Ter119 (BioLegend, clone TER-119, lot B286863); anti-CD34 (BD Biosciences, clone RAM34, lot 9015677); anti-CD16/32 (BD Biosciences, clone 2.4G2, lot 9133882); anti-Kit (BD Biosciences, clone 2B8, lot 8239973); anti-Sca1 (BioLegend, clone D7, lot B301237); anti–Gr-1 (BioLegend, clone RB6-8C5, lotB288472); anti-CD19 (BioLegend, clone 6D5, lot B248057); anti-CD49b (BioLegend, clone DX5, lot B272165); anti-90.2 (BioLegend, clone 53-2.1, lot B274408); anti-CD11c (BioLegend, clone N418, lot B262129); anti–IL-7Ra (BD Biosciences, clone SB/199, lot 9275199); anti–Ly-6G (BD Biosciences, clone 1A8, lot 9058981); anti-CD115 (BioLegend, clone AFS98, lot B268245); anti–Ly-6C (BioLegend, clone HK1.4, lot B268312); anti-CD40L (BioLegend, clone MR1, lot B270104); anti-CD62P (Emfret, clone Wug.E9, lot FE); anti-SiglecF (BD Biosciences, clone E50-2440, lot 9197200); anti-CD123 (Miltenyi Biotec, clone REA114, lot 1320020550); anti-KI67 (Invitrogen, Thermo Fisher Scientific, clone SolA15, lot 2191034); and anti-CD138 (BioLegend, clone 281-2, lot B286625).

All antibodies were used at a 1:700 dilution. Viable cells were identified as unstained with DAPI (Invitrogen, Thermo Fisher Scientific, D1306) or with LIVE/DEAD Fixable Far Read Dead Cell Stain kit or LIVE/DEAD Fixable Aqua Dead Cell Stain kit (both from Invitrogen, Thermo Fisher Scientific). Cells were identified as MKs (CD41^+^CD42b^+^Lin1^–^CD45^+^Tom^+^) using Pf4-cre- B6.Cg-*Gt(ROSA)26Sor^tm14(CAG-tdTdTomato)Hze^*/J mice; MK-erythrocyte progenitors (MEPs) (CD45^+^CD34^+^CD1632^–^Lin2^–^Sca1^–^Kit^+^); LSK cells (CD45^+^CD34^+^Lin2^–^Sca1^+^Kit^+^); platelets (CD41^+^CD42b^+^CD45^–^); BM leptin receptor cells (CD45^–^CD31^–^Ter119^–^Tom^+^) using Lepr-Cre-B6.Cg-*Gt(ROSA)26Sor^tm14(CAG-tdTdTomato)Hze^*/J mice; BM endothelial cells (CD45^–^CD31^+^Ter119^–^); blood Ly-6C^hi^ monocytes (CD45^+^CD11b^+^Ly-6G^–^Ly-6C^hi^); blood neutrophils (CD45^+^CD11b^+^Ly-6G^+^); bronchoalveolar lavage (BAL) neutrophils (CD45^+^CD11b^+^Ly-6G^+^F4/80^–^); BAL alveolar macrophages (CD45^+^F4/80^+^CD11b^–^SiglecF^+^); platelet-neutrophil aggregates (CD45^+^CD11b^+^Ly-6G^+^CD41^+^); and platelet-Ly-6C^hi^ monocytes (CD45^+^CD11b^+^Ly-6G^–^Ly-6C^hi^CD41^+^). Lineages were defined as Lin1 (NK1.1, CD3, CD11b, F4/80, B220) and Lin2 (Gr-1, Ter119, CD11b, B220, CD19, CD49b, CD90.2, CD11c, Il-7Ra).

The following anti-human antibodies were used for flow cytometry: anti-CD41a (BD Pharmingen, clone HIP8, lot 9171960); anti-CD42b (BD Pharmingen, clone HIP1, lot 9247599); anti-CD45 (BioLegend, clone 2D1, lot B293149); anti-CD2 (STEMCELL Technologies, clone RPA-2.10, lot 1000018050); anti-CD11b (STEMCELL Technologies, clone ICRF44, lot 1000017695); anti-CD14 (Invitrogen, Thermo Fisher Scientific, clone 61D3, lot 2124592); anti-CD56 (STEMCELL Technologies, clone HCD56, lot SC07565); anti-CD235 (BioLegend, clone HIR2, lot B263799); anti-CD11c (Invitrogen, Thermo Fisher Scientific, clone3.9, lot 2087652); anti-CD3 (STEMCELL Technologies, clone UCHT1, lot 1000010685); anti-CD19 (STEMCELL Technologies, clone HIB19, lot 1000019049); anti-CD66b (BioLegend, clone G10F5, lot B215969); anti-CD16 (STEMCELL Technologies, clone 3G8, lot BX31455); anti-CD24 (Invitrogen, Thermo Fisher Scientific, clone eBioSN3, lot 1938843); and streptavidin (BioLegend, lot B267737). MKs were identified as CD45^+^Lin^–^ (CD2, CD11b, CD14, CD56, CD235, CD11c, CD3, CD19, Cd66b, CD16, CD24) CD41a^+^CD42b^+^.

### MK ploidy assay

After BM and splenic cell collection, cells were placed onto a BSA gradient to isolate MKs. After fixation using 0.5% formaldehyde on ice for 30 minutes, MKs were stained with a solution containing 2 mM MgCl_2_, 0.005% saponin, 10 mg/mL propidium iodide, and 10 IU/mL RNAse A for 2 hours at 4°C and immediately analyzed on an LSR II flow cytometer (BD Biosciences).

### Cell sorting

BM or splenic cells were stained for specific cell population identification and sorted using a FACS Aria III cell sorter (BD Biosciences).

### ELISAs

SCF and CXCL12 from serum and BM fluid were measured using ELISA kits (MCK00 from R&D Systems and MCX120 from R&D Systems, respectively). Briefly, to collect BM fluid, a small hole was made at the tip of the long bones before spinning the BM out of the bones by centrifugation at 6000*g* for 6 minutes. Supernatant was then collected. IL-3 and TPO from serum was measured using ELISA kits (EK0403 from BosterBio and MTP00 from R&D Systems). IL-6 and TNF-α from plasma were measured using ELISA kits (M6000B from R&D Systems and MTA00B from R&D Systems, respectively). To quantify soluble NETs (citrullinated histone H3–DNA complexes), a custom ELISA was used. For antibody capture, an anti-citrullinated histone H3 antibody (ab5103, Abcam) was used. Detection of complexes was done using anti–DNA-HRP conjugate (Cell Death Detection ELISA^plus^ Kit, Roche; ref. 45).

### MK CFU assay

Five thousand sorted MEPs from BM or spleen were plated on MegaCult medium with cytokines (MegaCult-C Complete Kit with Cytokines, 04971, STEMCELL Technologies) following the manufacturer’s instructions. MK CFU were counted after 8 days in a 5% CO_2_ incubator at 37°C.

### PCR

Total RNA was isolated using the RNeasy Mini Kit (QIAGEN) or the RNeasy Micro Kit (QIAGEN) according to the manufacturer’s instructions. DNAse digestion, during RNA purification, was done using the RNase-free DNase Set (QIAGEN). RNA quantity and quality were measured using NanoDrop (Thermo Fisher Scientific). Total RNA (1 g) was taken for cDNA generation using iScript Reverse Transcription Supermix for RT-qPCR (Bio-Rad). Quantitative real-time TaqMan PCR was performed with the following TaqMan primers (Applied Biosystems): *Il6* (Mm00446190_m1); *Csf2* (Mm01290062_m1); *Il1b* (Mm00434228_m1); *Cxcl12* (Mm00445553_m1); *Il3* (Mm00439631_m1); *Kitl* (Mm00442972_m1); *Thpo* (Mm00437040_m1); and *Ccl5* (Mm01302427_m1). PCR was run on a Viia7 cycler, and gene expression was normalized to *GAPDH* and quantified with the 2ΔΔCt method.

### Preparation of mouse platelets for transfusion

As previously described (46, 47), blood was drawn from the inferior vena cava into a syringe containing acid citrate dextrose (MilliporeSigma, C3821). Platelet-rich plasma was obtained by mixing blood with modified HEPES-Tyrode buffer (140 mM NaCl, 2 mM KCl, 12 mM NaHCO_3_, 0.3 mM NaH_2_PO_4_, 1 mM MgCl_2_, 5.5 mM glucose, 5 mM HEPES, pH 6.8) containing 0.35% BSA, followed by centrifugation at 300*g* for 4 minutes. Prostacyclin (PGI_2_) was added to platelet-rich plasma (PRP) (500 nM final concentration), and platelets were then pelleted by centrifugation at 1000*g* for 6 minutes. Pelleted platelets were resuspended in modified HEPES-Tyrode buffer (pH 7.38) containing the adenosine diphosphate (ADP) scavenger apyrase (adenosine-5′-triphosphate diphosphohydrolase, 0.02 IU/mL final) before being rested for 45 minutes at 37°C. For platelet transfusions, recipient mice were injected i.v. with 3 × 10^8^ platelets, and BSA was added to a final concentration of 150 mg/g body weight of mice.

### Bacterial colony-forming assays

Whole blood samples were diluted, plated on tryptic soy agar (BD DIFCO), and incubated at 37°C. The number of bacterial colonies was counted 12–14 hours after incubation.

### Intravital imaging

2PIVM was used to observe MKs and platelet production in real time in mice (1, 48). Mice were anesthetized with ketamine and xylazine and secured with surgical tape to a custom heated microscope stage. A tracheal cannula was inserted and attached to a MiniVent mouse ventilator (Harvard Apparatus). Mice were ventilated with a stroke volume of 10 μL air (21% O_2_) per gram of mouse body weight, with a respiratory rate of 125 breaths per minute and a positive-end expiratory pressure of 2–3 cm H_2_O. Isoflurane (1%) was delivered continuously to maintain anesthesia. For spleen imaging, a skin incision was made on the left flank and along the costal margin to expose and externalize the spleen. A custom stainless-steel window (Supplemental Figure 2B) was fitted with an 8 mm round coverslip and applied to the spleen. For gentle immobilization of the preparation, 20–25 mmHg of suction was applied using a vacuum regulator (Amvex), and the window was secured to the stage using a set of 2 optical posts and a 90 degree angle post clamp (Thorlabs). A 2-photon microscope objective (CFI75 Apochromat 25XC W 1300, Nikon) was then lowered into place over the window for imaging. For BM 2PIVM, the calvarium was exposed at the coronal suture of the frontal bone. The imaged region was stabilized by a 3D printed apparatus that was affixed to the mouse skull with Vetbond and attached to the heated stage below, as described previously (1).

Intravital imaging was performed with a Nikon A1R Multi-photon microscope equipped with a Mai Tai DeepSee IR Laser (Spectra Physics) that was tuned to 950 nm for excitation of GFP and tdTdTomato. Emitted light was detected using a 25× water lens (Nikon) with green (500–550 nm) and red (570–620 nm) filters. Spleen images were captured with a high-resolution resonant scanner (512 × 512) and BM images using a high-resolution galvano scanner (512 × 512). The microscope was controlled using NIS Element AR software (5.11). A 352.256 μm × 336.384 μm x–y BM surface area (0.1185 mm^2^) was captured, and *Z*-stack images were acquired with total *Z*-depths of 10 μm. A 972.135 μm × 699.792 μm x–y spleen surface area (0.6803 mm^2^) was captured, and *Z*-stack images were acquired with total *Z*-depths of 10 μm. Images were analyzed using Imaris 9.5.1 software.

### Tyrosine hydroxylase staining

For tyrosine hydroxylase staining (25), freshly dissected femurs from mice were fixed in PBS and 2% PFA overnight at 4°C. After 2 washes with PBS, the bones were incubated in PBS and 30% sucrose overnight at 4°C. The bones were embedded in OCT (Thermo Fisher Scientific) and frozen. The OCT blocks were longitudinally cut with a cryostat (Leica) and washed several times to remove the OCT. The bones were placed into a 1.5 mL Eppendorf tube and were on rotation in the dark at room temperature (RT) for the staining process. The bones were first placed in blocking buffer (PBS plus 5% donkey serum, MilliporeSigma), 10% dimethyl sulfoxide (Santa Cruz Biotechnology), 0.5% IgePal630 (MilliporeSigma), and 1% BlokhenII (Aves Labs)) overnight at RT. The bones were then stained for 3 days in staining buffer (PBS, 5% donkey serum, 10% dimethyl sulfoxide, 0.5% IgePal630) with anti–tyrosine hydroxylase antibody (1:500, rabbit anti-mouse antibody, MilliporeSigma, catalog AB152) and washed multiple times with PBS for an entire day. The bones were then stained in staining buffer with a secondary antibody (1:300, donkey anti–rabbit IgG [H+L] antibody, Alexa Fluor 546, Thermo Fisher Scientific, A10040) for 3 days and washed around multiple times for an entire day with PBS. Next, the samples were stained with DAPI (1:2000, MilliporeSigma) in PBS for 30 minutes and washed twice with PBS for 30 minutes for each washing. Images were acquired using a Leica SP5 confocal microscope and analyzed with ImageJ software (NIH). To quantify neural fibers, the total TH^+^ area was normalized to the total DAPI-labeled BM area.

### Human spleen immunofluorescence

Human spleen samples from organ donors were fixed in 1% formaldehyde for 2 hours, cryoprotected with 30% sucrose overnight, and frozen in OCT. Cryosections (100 μm thick) were stained with primary antibodies (anti-CD42b, clone SP219, Abcam and anti-CD45, clone H130, BioLegend) in PBS with 0.3% Triton X-100, 0.2% BSA, and a 10% secondary host serum overnight. After washing, the sections were incubated with a secondary antibody (anti–rabbit IgG, AF488, Jackson ImmunoResearch) in PBS with 0.3% Triton X-100 before washing and mounted with VECTASHIELD (Vector Laboratories). Images were acquired using a Nikon A1R confocal microscope (UCSF Biological Imaging Development Center [BIDC]).

### Histology

Mouse femurs and spleens were fixed in 10% formalin for 24 hours. The femurs and spleens were then processed, paraffin wax embedded, sectioned and stained with H&E for analysis. Images were acquired with an Axio ScanZ.1 microscope (Zeiss).

### Platelet, neutrophil, and MRSA coincubations

BM cells were harvested in RT PBS without calcium. RBCs were eliminated after incubation of the pellet with 4 mL of 0.2% NaCl for 30 seconds. Cells were layered over 62% Percoll and centrifuged (1000*g* without braking for 30 minutes at RT). Neutrophils were recovered from the cloudy pellet obtained. Purity was checked by cytospin and was greater than 90% in all experiments. Cells were resuspended in PBS and plated per well in 96-well plates. For NETs ELISA experiments, neutrophils (2.5 × 10^5^ neutrophils) were cultured at 37°C and 5% CO_2_ and stimulated with MRSA (1.25 × 10^6^ CFU) and platelets (2.5 × 10^7^ platelets) for 4 hours. For CFU experiments, neutrophils (5 × 10^5^ neutrophils) were cultured at 37°C and 5% CO_2_ and stimulated with MRSA (3 × 10^7^ CFU) and platelets (1 × 10^8^ platelets) for 4 hours in DMEM. We used the SF8300 strain of MRSA (methicillin-resistant *S. aureus*, obtained from C. Chambers, UCSF), which is a minimally passaged USA300 clinical strain representative of the epidemic clone USA300-0114 (45). Cell-free supernatant was used to measure NETs by ELISA and supernatant for MRSA CFU capacities using tryptic soy agar (BD DIFCO).

### NETs in vitro assay

BM cells were harvested in RT PBS without calcium. RBCs were eliminated after incubation of the pellet with 4 mL 0.2% NaCl for 30 seconds. Cells were layered over 62% Percoll and centrifuged (1000*g* without braking for 30 minutes at RT). Neutrophils were recovered from the cloudy pellet obtained. Purity was checked by cytospin and was greater than 90% in all experiments. Cells were resuspended in PBS and plated in 96-well plates. Neutrophils (2.5 × 10^5^ neutrophils) were cultured at 37°C and 5% CO_2_ and stimulated with 200 ng/mL mouse recombinant CD40L (R&D Systems) or with 100nM PMA (MilliporeSigma) for 4 hours. Cell-free supernatant was used to measure NETs by ELISA.

### RNA-Seq analysis

For RNA-Seq experiments, we used Pf4-TdTomato mice and sorted CD41^+^CD42b^+^Lin1^–^CD45^+^Tom^+^ cells from BM and spleen, 5 days after saline treatment or sepsis, and the cells were prepared for single-cell suspension and flow cytometric analysis. Three independent replicates were used for each population. Total mRNA was isolated from 300 purified cells using the SMART-Seq HT kit (Takara Bio). An mRNA library was prepared by the UCSF Functional Genomics Core using the Nextera XT DNA Library Prep kit (Illumina).

Using log ratio and fold-change cutoffs (FDR <0.05), we found 776 genes that were differentially expressed in the saline condition: 275 were upregulated in BM MKs and 501 in splenic MKs. In the sepsis condition, we found 789 genes that were differentially expressed: 306 were upregulated in BM MKs and 483 in splenic MKs. Functional pathways representative of each gene signature were analyzed for enrichment in gene categories from the Gene Ontology Biological Processes (GO-BP) database (Gene Ontology Consortium) using Enrichr bioinformatics resources (49, 50). GO-BP categories with at least 3 genes and *P* values of less than 0.001 were identified. The BioVenn diagram was created using DeepVenn software (www.deepvenn.com; ref. 51).

### Data availability

The RNA-Seq data supporting the findings of this study have been deposited in the NCBI’s Gene Expression Omnibus (GEO) database (GEO GSE196607).

### Statistics

All data are presented as the mean ± SEM. The *n* value represents the number of mice in each experiment, as detailed in the figure legends. Statistical significance was determined using an unpaired, 2-tailed Student’s *t* test to compare 2 groups or a 1- or 2-way ANOVA for multiple-group comparisons. Statistical analyses were performed using GraphPad Prism 7 (Graphpad Software) and FlowJo 10.7 software (BD Biosciences). *P* values of 0.05 or less were considered statistically significant, as indicated by asterisks in the figures, unless otherwise noted.

### Study approval

All experiments conformed to ethical principles and guidelines approved by the IACUC of UCSF.

## Author contributions

CV designed and conducted most of the experiments, analyzed the data, and wrote the manuscript. MM, SJC, KMW, SR, EG, CC, and YS performed experiments. LQ performed the spleen transplantation experiments. RB analyzed data. DRC and JRG provided human spleen samples. SJC, ADL, EP, SMF, and FKS provided intellectual input and edited the manuscript. MRL designed, conducted experiments, analyzed data, and wrote the manuscript.

## Supplementary Material

Supplemental data

Supplemental table 1

Supplemental table 2

Supplemental video 1

Supplemental video 2

Supplemental video 3

Supplemental video 4

Supplemental video 5

## Figures and Tables

**Table 1:**
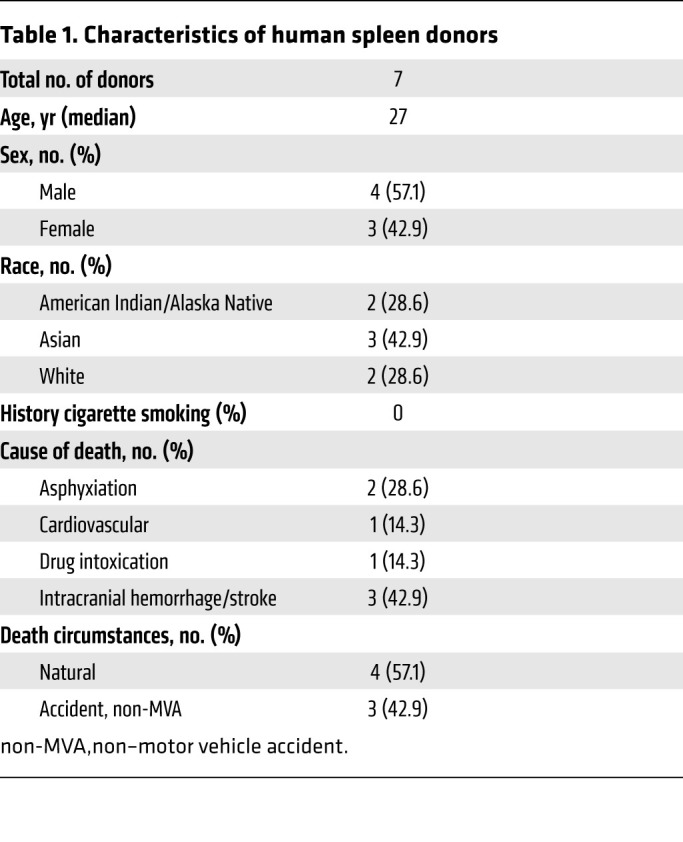
Characteristics of human spleen donors

## Version 1 02/22/2022

In-Press Preview

## Version 2 04/01/2022

Electronic publication
