# Supplementary material for: Sepsis promotes splenic production of a protective platelet pool with high CD40 ligand expression
Source: J Clin Invest. 2022 Apr 1;132(7):e153920. doi: 10.1172/JCI153920 (PMC8970674; doi:10.1172/JCI153920)
Supplement: Supplemental data [file jci-132-153920-s143.pdf]

Video 1. Spleen 2PIVM in PF4-mTmG mice 5 days after saline or CS injection. The video shows large extravascular MKs and also MKs releasing proplatelets (arrows) in the spleen of mice 5 days after saline or CS injection. Scale bar and acquisition time are indicated. A Gaussian filter was applied during analysis.

Video 2. Calvarium BM 2PIVM in PF4-mTmG mice 5 days after saline or CS injection. The video shows large extravascular MKs and also MKs releasing proplatelets (arrows) in the BM of mice 5 days after saline or CS injection. Scale bar and acquisition time are indicated. A Gaussian filter was applied during analysis.

Video 3. Z-stack video of MKs (green) and MKs releasing proplatelets (arrow) in human spleen. Scale bar is indicated. 1st example is 21 μm depth; 2nd example is 14 μm depth; 3rd example is 13 μm depth; 0.5 μm steps.

Video 4. Spleen 2PIVM in PF4-mTmG mice 5 days CS injection and treatment with daily anti-HRP or anti-IL-3 antibody. The video shows large extravascular MKs and MKs releasing proplatelets (arrows) in the spleen of mice 5 days CS injection and treatment with daily anti-HRP or anti-IL-3 antibody. Scale bar and acquisition time are indicated. A Gaussian filter was applied during analysis.

Video 5. Calvarium BM 2PIVM in PF4-mTmG mice 5 days CS injection and treatment with daily anti-HRP or anti-IL-3 antibody. The video shows large extravascular MKs and MKs releasing proplatelets (arrows) in the BM of mice 5 days CS injection and treatment with daily anti-HRP or anti-IL-3 antibody. Scale bar and acquisition time are indicated. A Gaussian filter was applied during analysis.

Table 1. Characteristics of human spleen donors.

Table 2. RNA-sequencing of BM MKs versus Spleen MKs 5 days after saline injection.

Table 3. RNA-sequencing of BM MKs versus Spleen MKs 5 days after CS injection.

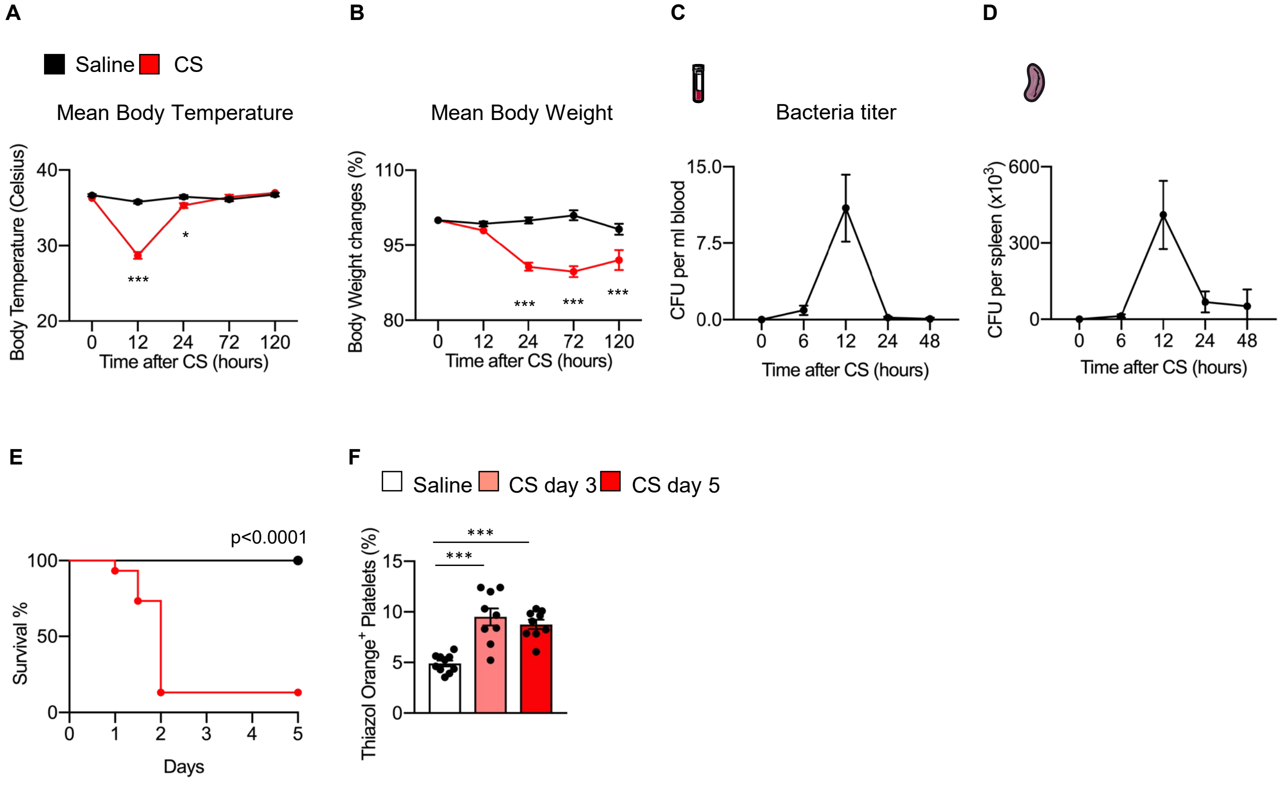

Supplemental Figure 1. Cecal slurry model of sepsis increases platelet production. (**A**) Mean body temperature and (**B**) body weight change following saline or CS injection (200 µl). n=9, 10 mice, respectively. Bacterial titer in blood (**C**) and spleen (**D**) after CS injection. n=5, 18 mice, respectively. (**E**) Kaplan-Meier survival curve in mice receiving saline or CS lethal injection (400 µl). n=15 mice per group. (**F**) Percentage of thiazol orange positive platelets. n=10, 9, 9 mice, respectively. Data are mean ± SEM. Significance was assessed using two-way ANOVA. *p<0.05, ***p<0.0001.

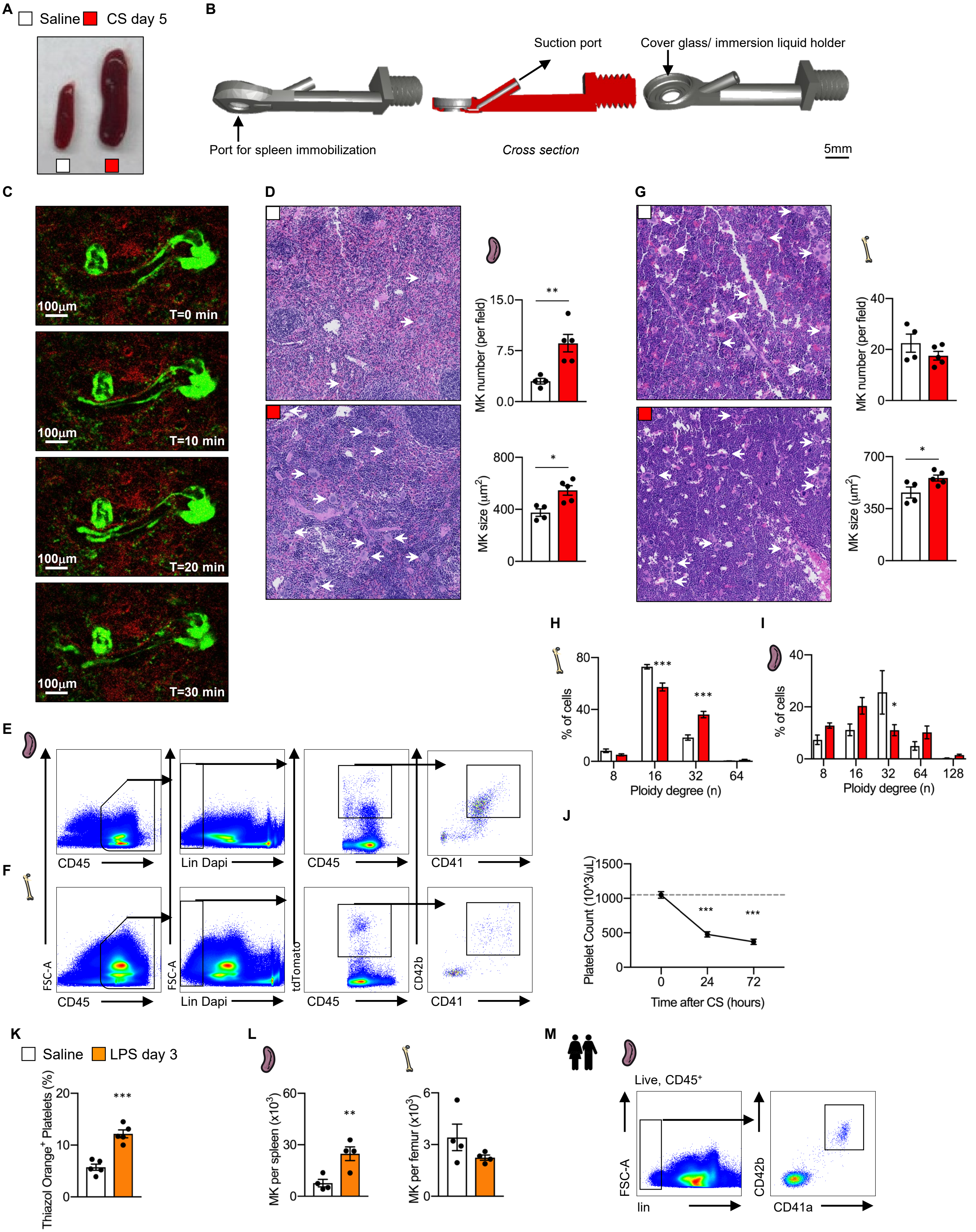

Supplemental Figure 2. Cecal slurry model of sepsis increases splenic MK content. (**A**) Representative image of the spleen 5 days after saline and CS injection. (**B**) Illustration of custom imaging window for spleen 2PIVM. (**C**) Sequential images show a large-sized MK (green) in the spleen (red) with proplatelet extensions. Scale bar = 100 μm. (**D**) H&E staining and analysis of MK number and size in the spleen 5 days after saline and CS injection. n=4, 5 mice, respectively. Gating strategy for MKs in the spleen (**E**) and the BM (**F**). (**G**) H&E staining and analysis of MK number and size in the BM 5 days after saline and CS injection. n=4, 5 mice, respectively. Polyploidy analysis of native MKs in the BM (**H**) and spleen (**I**) 5 days after saline and CS injection. n=10, 8 mice, respectively. (**J**) Mean platelet count after LPS injection. n=5 mice. (**K**) Percentage of thiazol orange positive platelets. n=5 mice. (**L**) Enumeration of MKs in the spleen and BM 3 days after Saline or LPS injection. n=4 mice. (**M**) Flow cytometric analysis of MKs in the human spleen. Data are mean ± SEM. Significance was assessed using two-tailed unpaired Student's *t*-test and two-way Anova. *p<0.05, **p<0.005, ***p<0.0001.

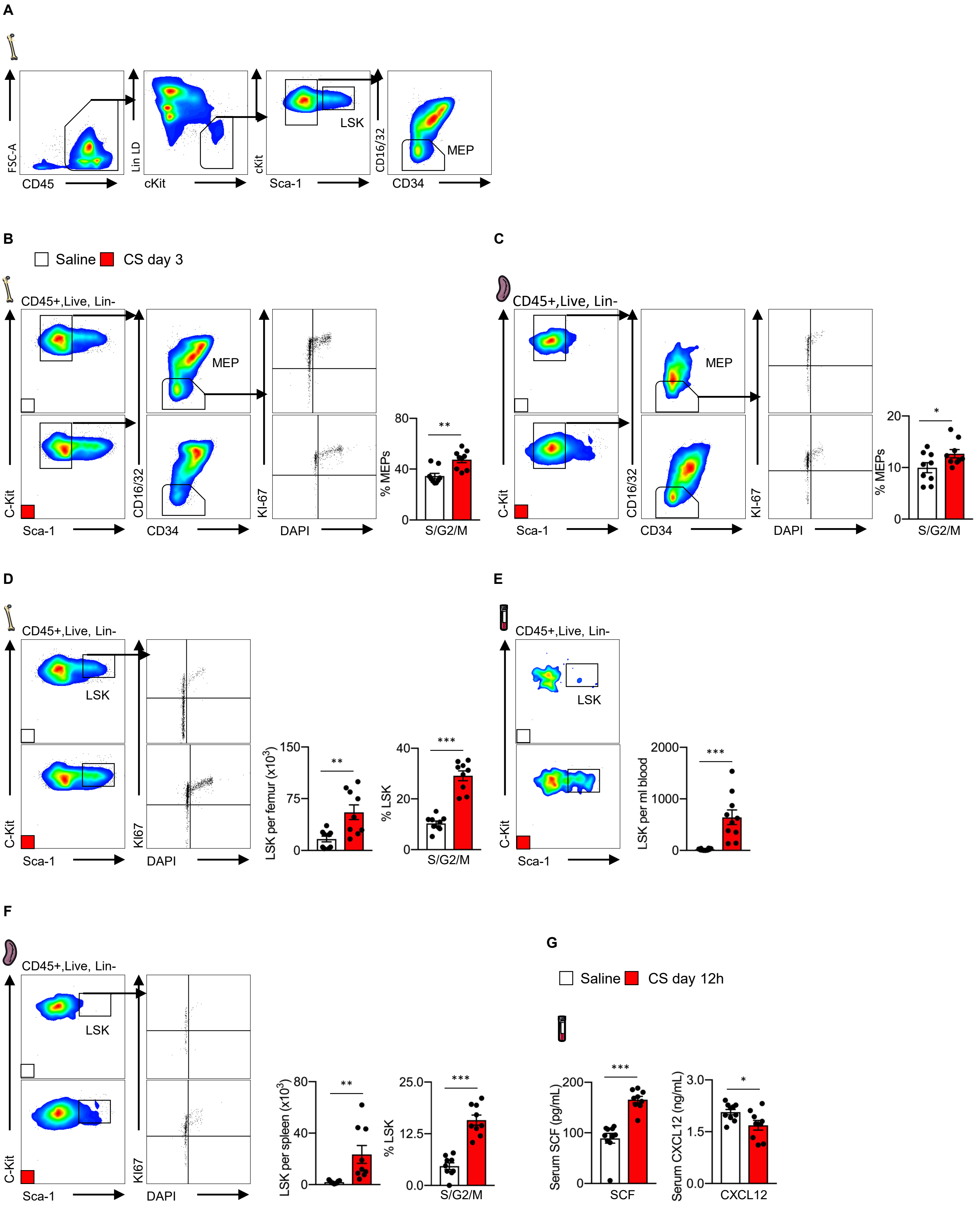

Supplemental Figure 3. Increased levels of LSKs in the spleen after sepsis. (**A**) Gating strategy for MEP and LSK. Analysis and proliferation rate of MEPs in the BM (**B**) and the spleen (**C**) 3 days after saline and CS injection. n=9 mice per group. Analysis, enumeration, and proliferation rate of LSKs in the BM (**D**), blood (**E**), and spleen (**F**) at 3 days after saline and CS injection. n=9, 10 mice, respectively. (**G**) Blood SCF and CXCL12 levels 12 hours after saline and CS injection. n=10, 9 mice, respectively. Data are mean ± SEM. Significance was assessed using two-tailed unpaired Student's *t*-test or two-way ANOVA. *p<0.05, **p<0.005, ***p<0.0001.

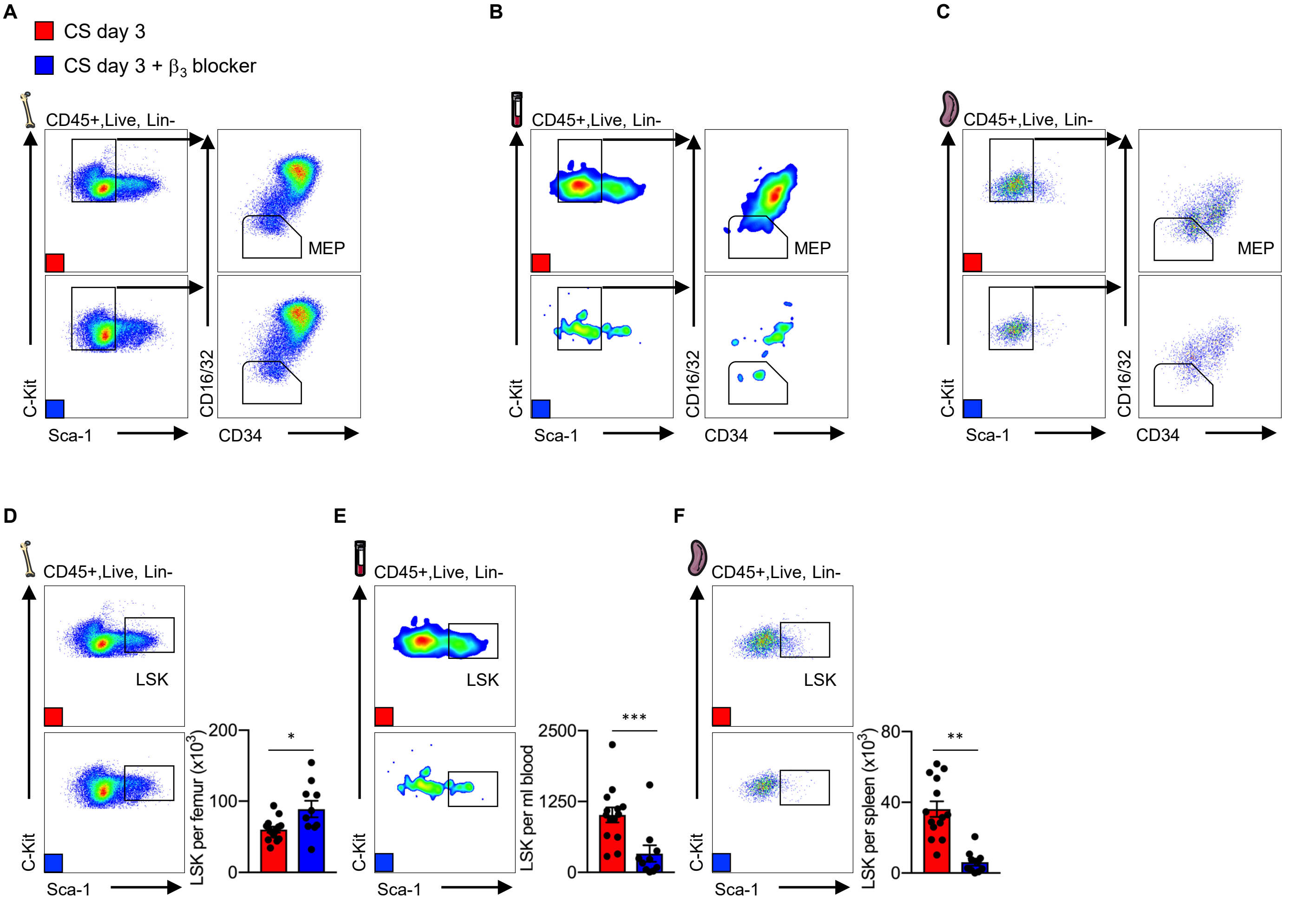

Supplemental Figure 4. β3 adrenergic receptor blockade reduces splenic LSK content after sepsis. Analysis of MEPs in the BM (**A**), blood (**B**) and spleen (**C**) 3 days after CS injection and treatment with DMSO or β3 antagonist twice daily. Analysis and enumeration of LSKs in the BM (**D**), blood (**E**) and spleen (**F**) 3 days after CS injection and treatment with DMSO or β3 antagonist twice daily. n=14, 10 mice, respectively. Data are mean ± SEM. Significance was assessed using two-tailed unpaired Student's *t*-test or two-way ANOVA. *p<0.05, **p<0.005, ***p<0.0001.

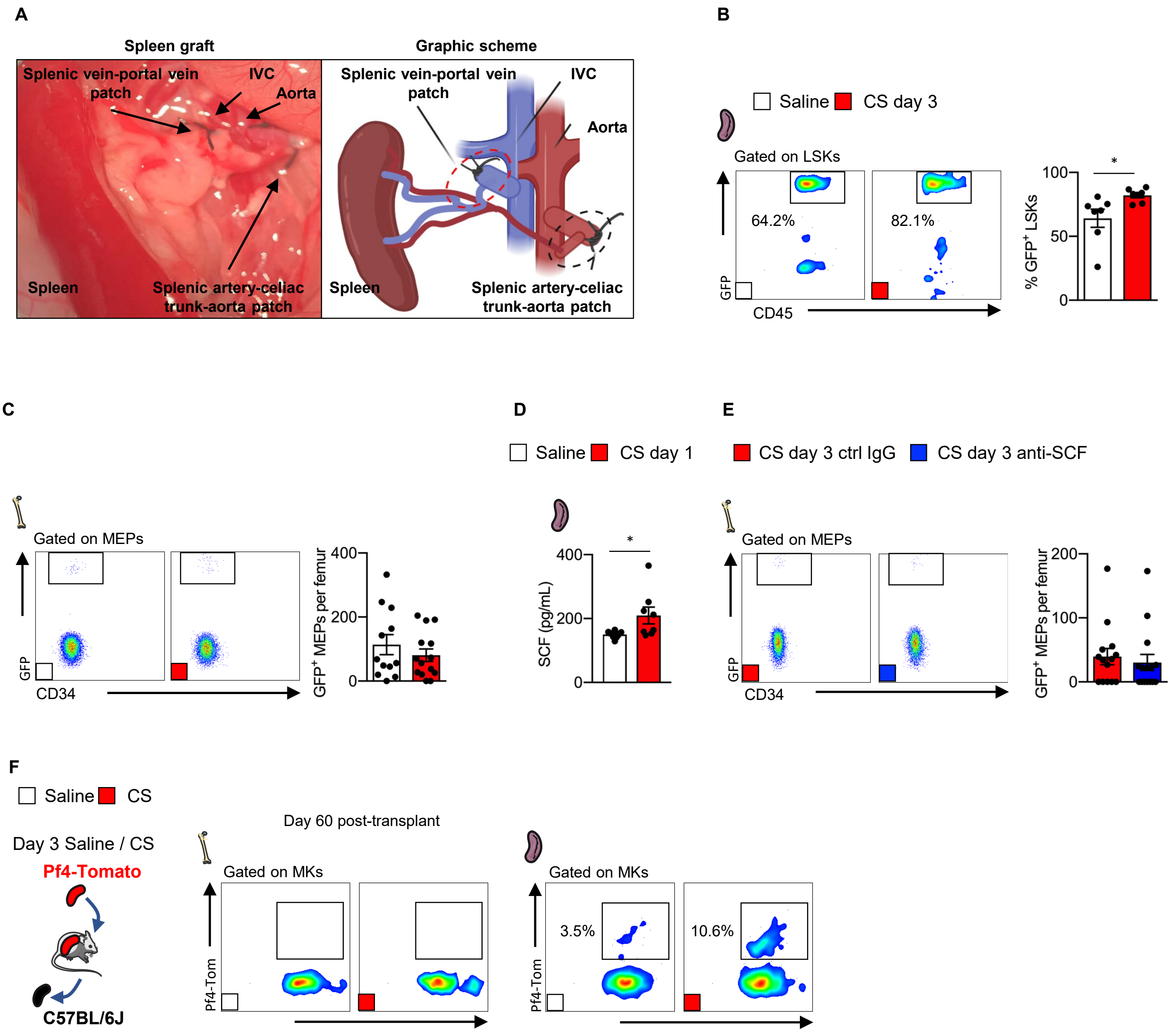

Supplemental Figure 5. Splenic engraftment and niche experiments. (**A**) Representative image and schematic of mouse spleen transplantation protocol. (**B**) Analysis and enumeration of GFP⁺ LSKs in the spleen 5 days after saline and CS injection. n=5 mice. (**C**) Analysis and enumeration of adoptively transferred GFP⁺ MEPs in the BM 3 days after saline and CS injection. n=12, 14 mice, respectively. (**D**) Spleen SCF levels 1 day after saline and CS injection. n=10, 8 mice, respectively. (**E**) Analysis and enumeration of adoptively transferred GFP⁺ MEPs in the BM 3 days after CS injection and treatment with IgG or SCF neutralizing antibody. n=13, 14 mice, respectively. (**F**) Flow cytometric analysis of tomato⁺ MKs in the BM (left) and spleen (right) 60 days after transplantation of a donor spleen obtained 3 days after saline and CS injection. n=4, 5 mice, respectively. Data are mean ± SEM. Significance was assessed using two-tailed unpaired Student's *t*-test. *p<0.05.

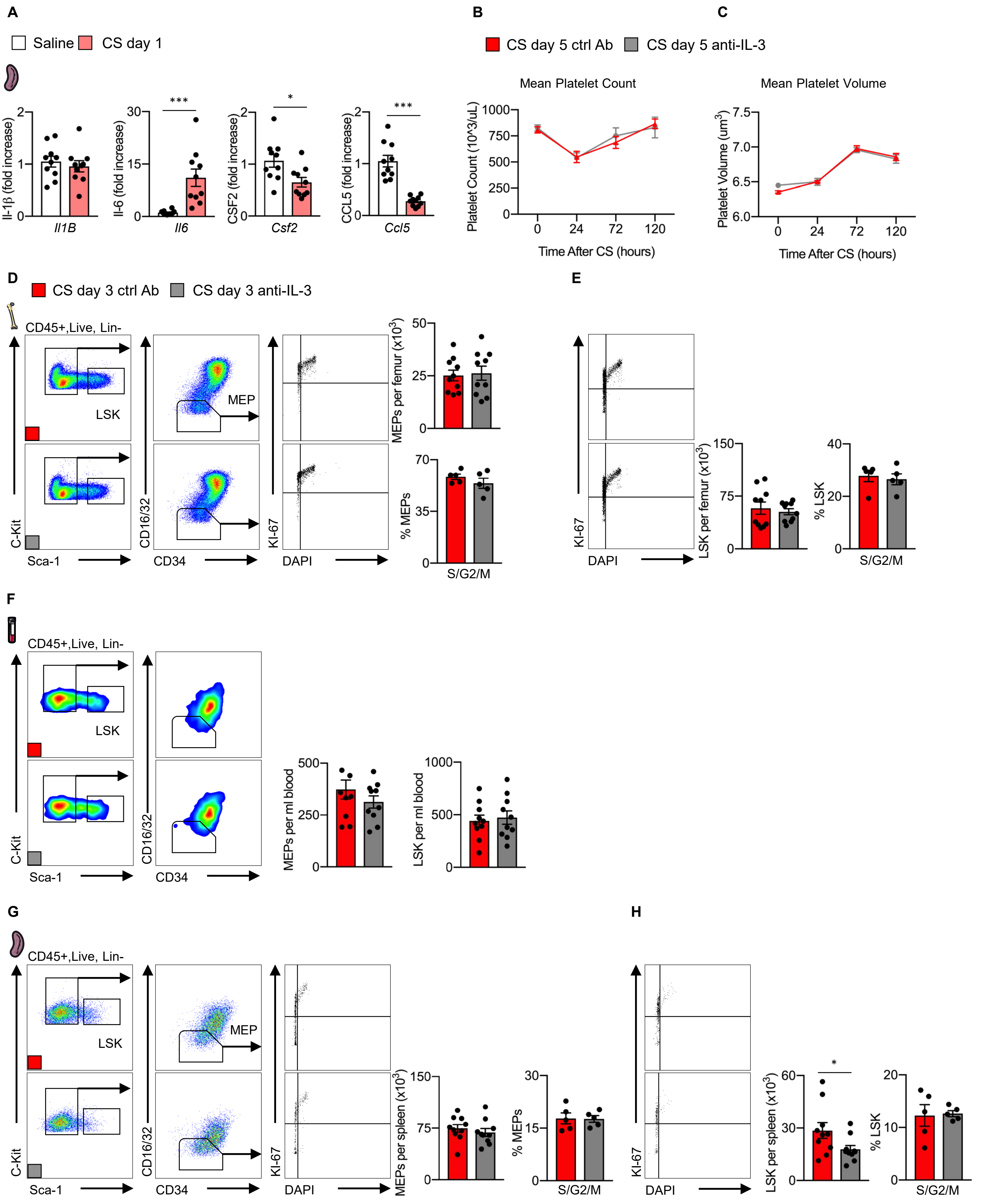

Supplemental Figure 6. IL-3 neutralization has no effect on hematopoietic progenitors after sepsis. (**A**) Expression of hematopoietic factors *IL1β*, *IL6*, *CSF2* and *CCL5* in the spleen as assessed by qPCR 1 day after saline and CS injection. n=10 mice. (**B**) Mean platelet count and (**C**) mean platelet volume after CS injection and treatment with daily anti-HRP or anti-IL-3 antibody. n=9, 7 mice, respectively. Analysis, enumeration and proliferation rates of BM MEPs (**D**) and LSKs (**E**) 3 days after CS injection and treatment with daily anti-HRP or anti-IL-3 antibody. n=10 and 5 mice, respectively. (**F**) Analysis and enumeration of blood MEPs and LSKs 3 days after CS injection and treatment with daily anti-HRP or anti-IL-3 antibody. n=10 mice per group. Analysis, enumeration, and proliferation rates of spleen MEPs (**G**) and LSKs (**H**) 3 days after CS injection and treatment with daily anti-HRP or anti-IL-3 antibody. n=10 and 5 mice, respectively. Data are mean ± SEM. Significance was assessed using two-tailed unpaired Student's *t*-test or two-way ANOVA. *p<0.05, ***p<0.0001.

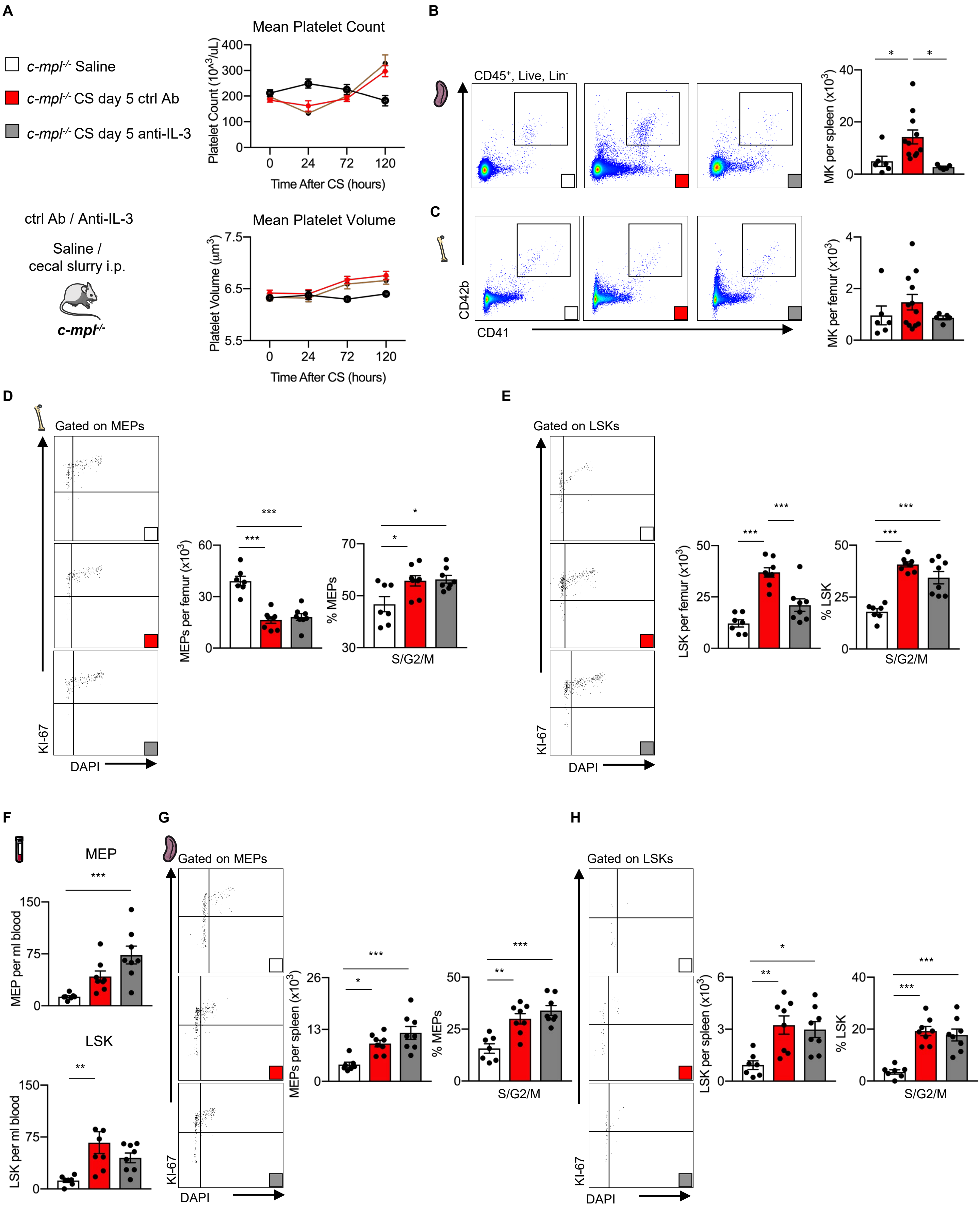

Supplemental Figure 7. Effect of IL-3 neutralization on hematopoietic progenitors in *c-mpl*-/- mice after sepsis. (**A**) Mean platelet count and mean platelet volume of *c-mpl*-/- mice after saline and CS injection and treatment with daily anti-HRP or anti-IL-3 antibody. n=9, 7 mice, respectively. Enumeration and analysis of MKs in the spleen (**B**) and BM (**C**) of *c-mpl*-/- mice at 5 days after saline and CS injection and treatment with anti-HRP or anti-IL-3 antibody. n=6, 11, 5 mice, respectively. Enumeration and proliferation rates of BM MEPs (**D**) and LSKs (**E**) 5 days after saline and CS injection and treatment of *c-mpl*-/- mice with anti-HRP or anti-IL-3 antibody. n=7, 8, 8 mice, respectively. (**F**) Enumeration of blood MEPs and LSKs 5 days after saline and CS injection and treatment of *c-mpl*-/- mice with anti-HRP or anti-IL-3 antibody. n=7, 8, 8 mice, respectively. Enumeration and proliferation rates of spleen MEPs (**G**) and LSKs (**H**) 5 days after saline and CS injection and treatment of *c-mpl*-/- mice with anti-HRP or anti-IL-3 antibody. n=7, 8, 8 mice, respectively. Data are mean ± SEM. Significance was assessed using one-way ANOVA. *p<0.05, **p<0.005, ***p<0.0001.

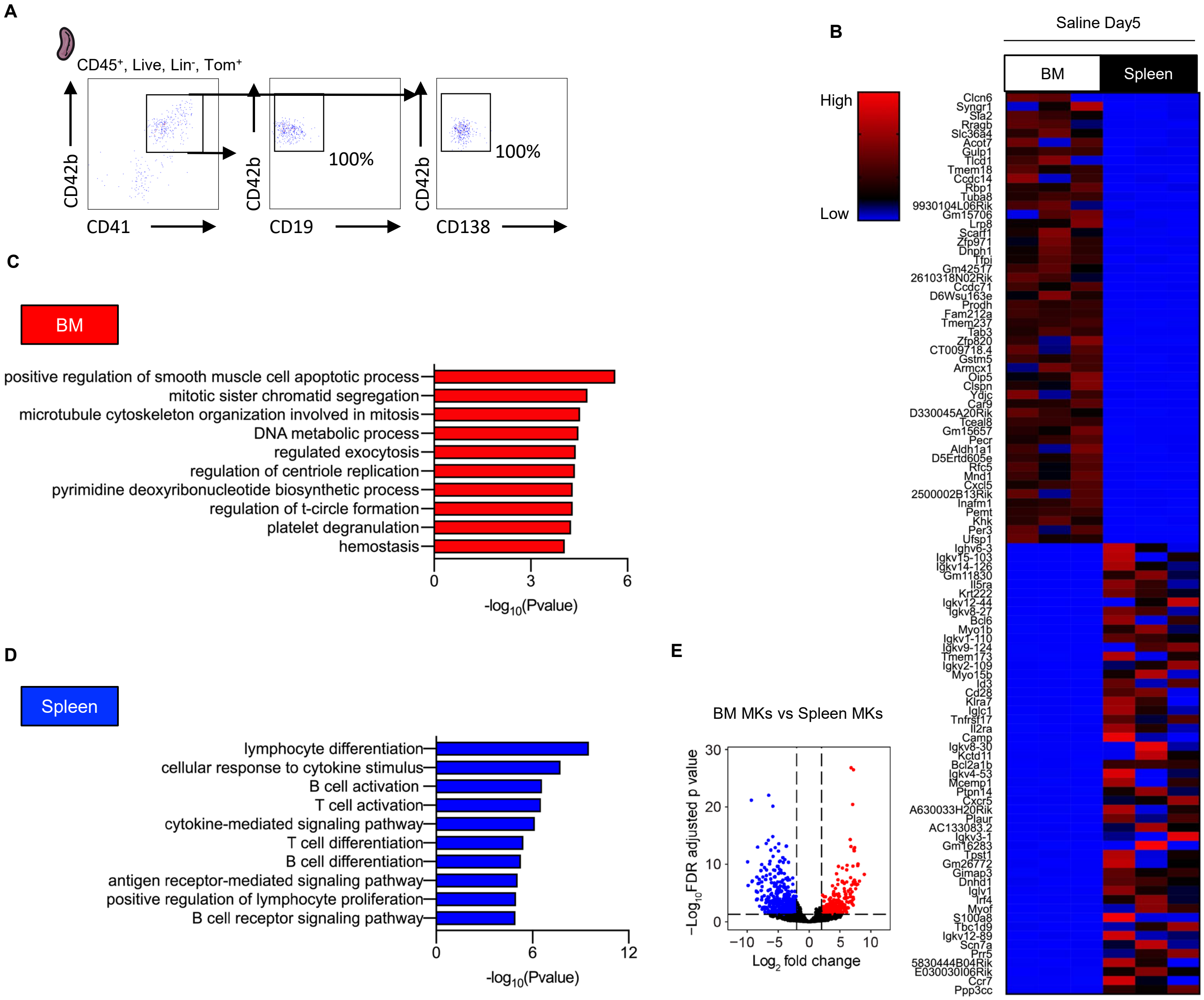

Supplemental Figure 8. BM and spleen MK RNA-seq. (**A**) Flow cytometry strategy for sorting of spleen MKs for RNA seq. (**B**) MKs were sorted from PF4-tom BM and spleen 5 days after saline followed by mRNA isolation and sequencing. Relative mRNA expression from low (blue) to high (red) of the top 50 genes differentially increased in the BM versus spleen (upper part) and the top 50 genes differentially increased in the spleen versus BM (lower part). (FDR<0.05) (**C, D**) Gene ontology biological processes analysis related to genes upregulated in BM MKs (**C**) or upregulated in spleen MKs (**D**) 5 days after saline injection. Top 10 biological processes are shown. (**E**) Volcano plots of DEG fold change between BM MKs and spleen MKs in saline control. n=3 mice.

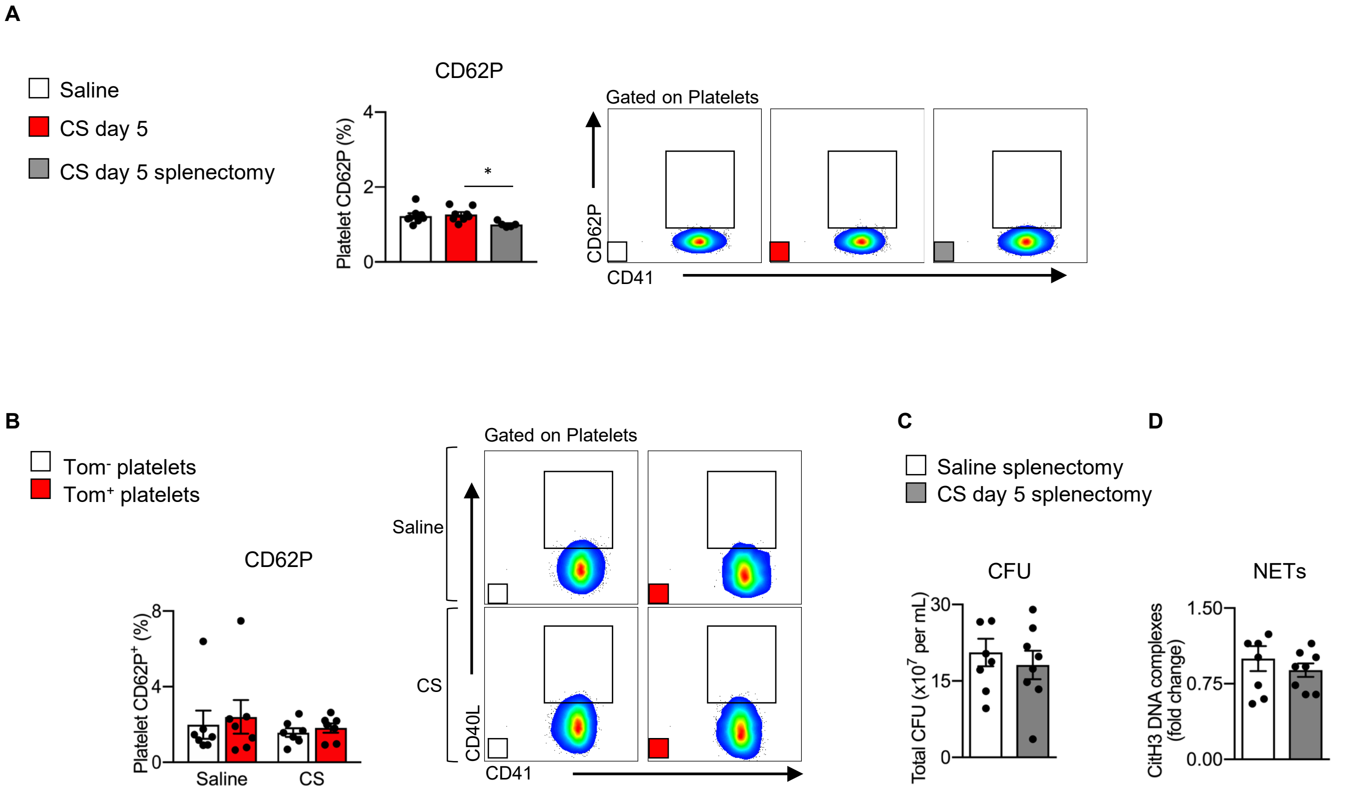

Supplemental Figure 9. Spleen-derived platelet and effects of splenectomy on platelet phenotype and function. (**A**) Percentage and analysis of $CD62P^+$ platelets 5 days after saline, CS injection, and CS injection in splenectomized mice. n=8, 8, and 5 mice, respectively. (**B**) Percentage of $CD62P^+$ donor-derived tomato$^+$ platelets. n=7 mice per group. CFU counts (**C**) and CitH3-DNA complexes (**D**) after neutrophil, MRSA, and platelets (obtained from 5 days after saline or CS injection in splenectomized mice) coincubation. n=7 and 8 mice, respectively. Data are mean ± SEM. Significance was assessed using one-way ANOVA. $^*p<0.05$.

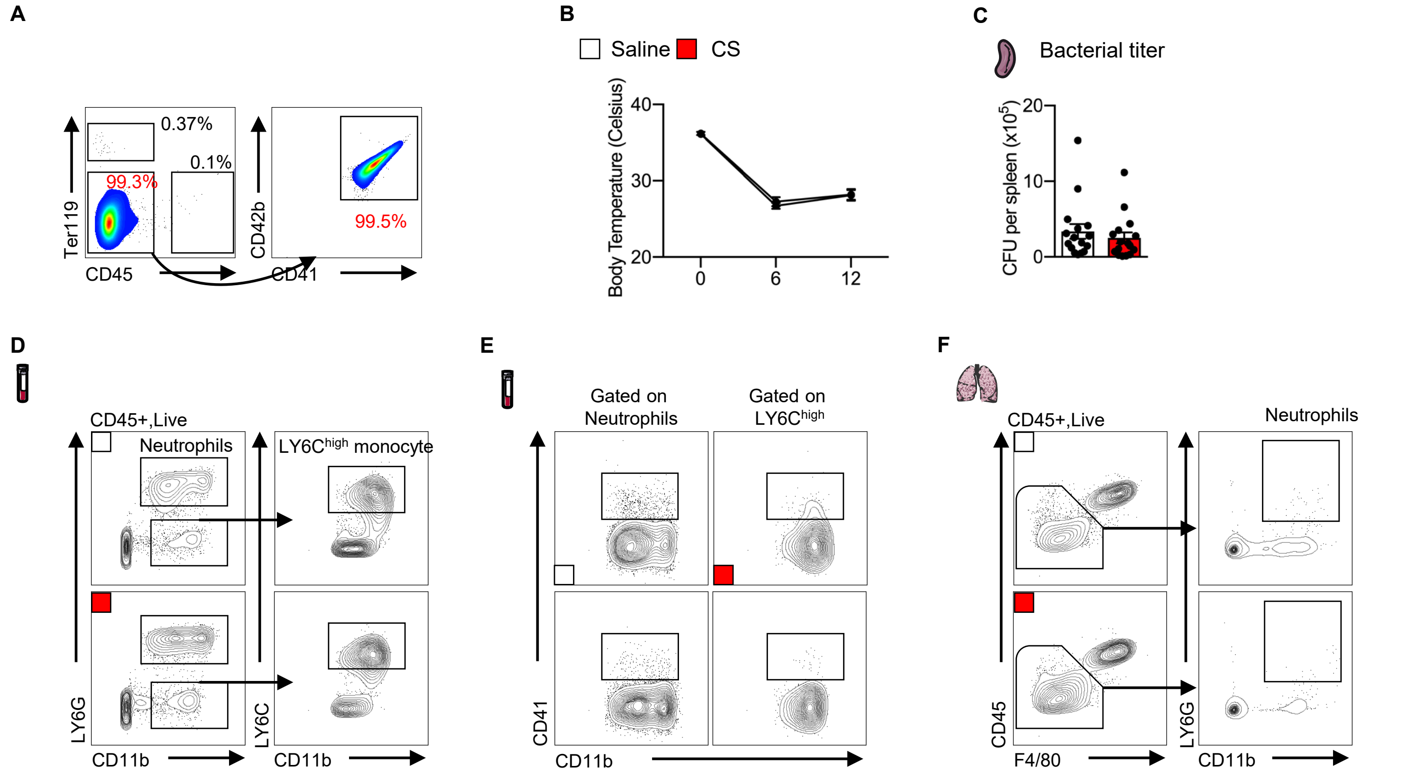

Supplemental Figure 10. Platelet transfusion experiments. (**A**) Analysis of isolated platelets. Septic mice were transfused with isolated platelets from day 5 post-saline or CS injection and tested for (**B**) mean body temperature and (**C**) splenic bacterial titer. n= 15 and 16 mice per group, respectively. (**D**) Analysis of neutrophils and Ly6C^{high} monocytes in blood. (**E**) Analysis of platelet-neutrophil and platelet-Ly6C^{high} monocyte aggregates in blood. n=15, 16 mice per group, respectively.(**F**) Analysis of BAL neutrophils of septic mice transfused with isolated platelets from day 5 post-saline or CS injection. n= n= 15 and 16 mice per group, respectively. Data are mean ± SEM.

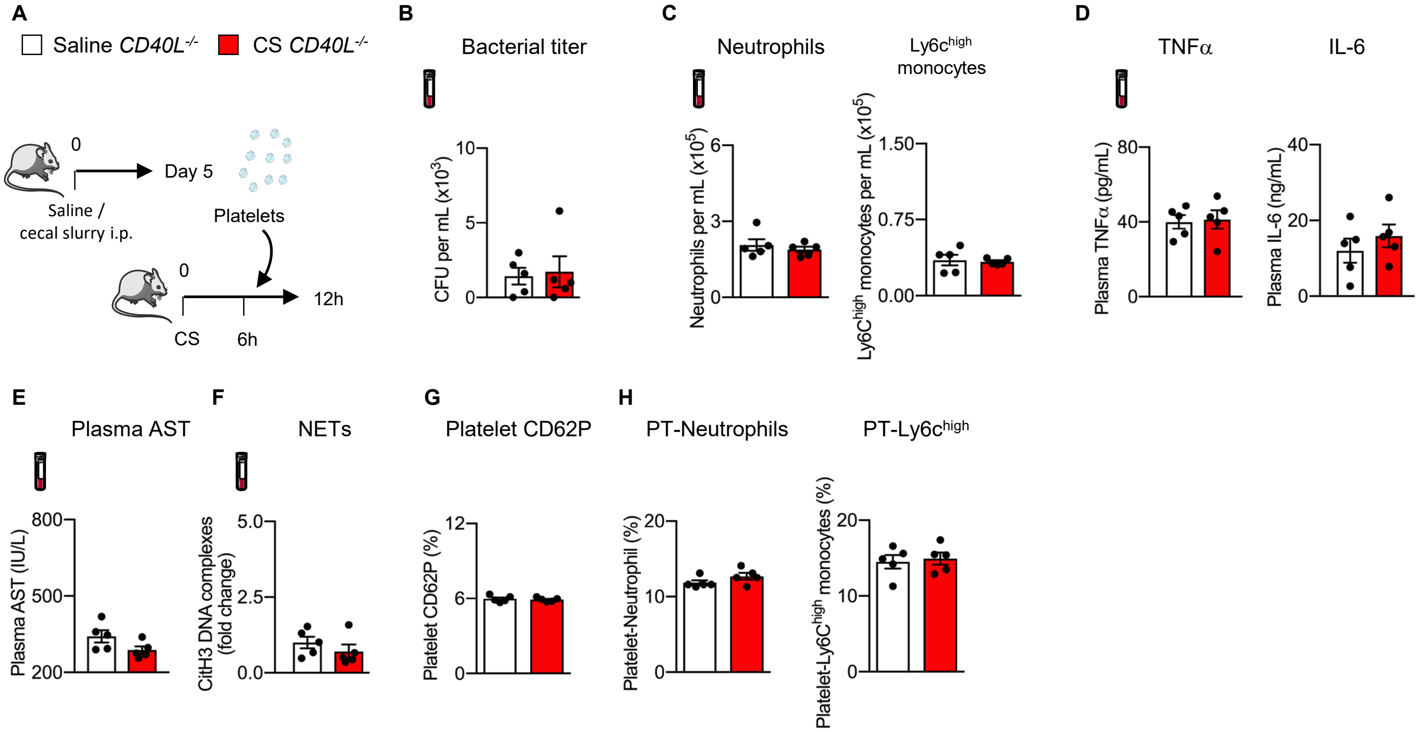

Supplemental Figure 11. ***CD40L*$^{-/-}$ platelet transfusion experiments.** (**A**) Schematic of septic mice transfused with *CD40L*$^{-/-}$ platelets isolated from either day 5 post-saline or CS injection experiments. (**B**) Bacterial CFU in the blood. (**C**) Enumeration of neutrophils and Ly6C^{high} monocytes in blood. (**D**) TNFα and IL-6 plasma measurements. (**E**) Aspartate aminotransferase plasma measurements (**F**) CitH3-DNA complexes (NETs) in the blood. (**G**) Enumeration of CD62P^{+} platelets. (**H**) Enumeration of neutrophil-platelet and LY6C^{high} monocytes-platelet aggregates in the blood. n=5 mice per group. Data are mean ± SEM.
